# LIF Promotes Sec15b‐Mediated STAT3 Exosome Secretion to Maintain Stem Cell Pluripotency in Mouse Embryonic Development

**DOI:** 10.1002/advs.202407971

**Published:** 2024-10-30

**Authors:** Li Xu, Jinjun Ji, Lingbo Wang, Jieli Pan, Mingzhe Xiao, Chenxi Zhang, Yihong Gan, Guanqun Xie, Mingdian Tan, Xinchang Wang, Chengping Wen, Yongsheng Fan, Y. Eugene Chin

**Affiliations:** ^1^ College of Basic Medical Science Zhejiang Chinese Medical University 548 Binwen Road Hangzhou 310051 China; ^2^ Clinical Medicine Research Institute Zhejiang Provincial People's Hospital Hangzhou Medical College 158 Shangtang Road Hangzhou Zhejiang 310014 China; ^3^ Group of Epigenetic Reprogramming, State Key Laboratory of Cell Biology, Institute of Biochemistry and Cell Biology, Shanghai Institutes for Biological Sciences Chinese Academy of Sciences Shanghai 200031 China; ^4^ Key Laboratory of Stem Cell Biology Institute of Health Sciences Chinese Academy of Sciences Shanghai 200031 China; ^5^ Department of Rheumatology The Second Affiliated Hospital of Zhejiang Chinese Medical University Hangzhou 310005 China

**Keywords:** cell pluripotency, exosome, multivesicular endosomes (MVEs), STAT3, Sec15b

## Abstract

LIF maintains self‐renewal growth in mouse embryonic stem cells (mESC) by activating STAT3, which translocates into nucleus for pluripotent gene induction. However, the ERK signaling pathway activated by LIF at large counteract with pluripotent gene induction during self‐renewal growth. Here, it is reported that in mESC STAT3 undergoes multivesicular endosomes (MVEs) translocation and subsequent secretion, LIF‐activated STAT3 is acetylated on K177/180 and phosphorylated on Y293 residues within the N‐terminal coiled‐coil domain, which is responsible for the interaction between STAT3 and Secl5b, an exocyst complex component 6B (EXOC6B). STAT3 translocation into MVEs resulted in the downregulation of T202/Y204‐ERK1/2 phosphorylation and up‐regulation of S9‐GSK3β phosphorylation for maintaining mESC self‐renewal growth. STAT3 with K177R/K180R or Y293F substitution fails to execute MVEs translocation and Secl5b‐dependent secretion. Mice expressing K177RK180R substitution (STAT3*^mut/mut^*) are partially embryonic lethal. In STAT3*^mut/mut^* embryos, gene expressions related to hematological system function changed significantly and those living ones carry a series of abnormalities in the hematopoietic system. Furthermore, mice with Secl5b knockout exhibit embryonic lethality. Thus, Secl5b mediated STAT3 MVEs translocation regulates the balance of ERK and GSK3β signaling pathways and maintain mESC self‐renewal growth, which is involved in regulating the stability of hematopoietic system.

## Graphical abstract

LIF‐induced interaction between STAT3 and Sec15b promotes STAT3 translocation to MVEs and its subsequent secretion. This process regulates the intracellular balance of ERK and GSK3β pathways, thereby playing a crucial role in maintaining stem cell pluripotency. Disruption of this mechanism impairs embryonic development, particularly affecting hematopoiesis.

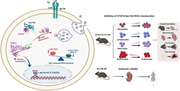

## 1 Introduction

Mouse embryonic stem cells (mESC) are typically obtained from the inner cell mass of pre‐implantation embryos and are defined by three unique characteristics: pluripotency, self‐renewal, and unlimited proliferation.^[^ ^1^ ^]^ mESC require the constant presence of leukemia inhibitory factor (LIF) to maintain their pluripotency properties.^[^ ^2^ ^]^ The STAT3 pathway activated by LIF has been recognized as pivotal for enhancing the pluripotency or self‐renewal capabilities of mESC.^[^ ^3^, ^4^ ^]^

Signaling pathways including the PI3K, ERK, and the STAT3 pathways are often activated simultaneously in cells by the LIF present.^[^ ^5^ ^]^ In the absence of LIF, artificial activation of STAT3 could be sufficient to support mESC pluripotency and self‐renewal growth.^[^ ^6^ ^]^ In STAT3‐deficient mESC, LIF is no longer capable of supporting pluripotency or self‐renewal. However, the application of ERK and GSK3β inhibitors (2i) can restore the ability for pluripotent growth. This observation suggests that STAT3 activation functions analogously to an inhibitor of ERK and GSK3β pathways.^[^ ^7^ ^]^ However, the relationship between STAT3 activation and differentiation restriction by LIF has not been established yet.

The maintenance of pluripotency in mESC necessitates the nuclear translocation of STAT3.^[^ ^4^ ^]^ Interestingly, Asrij, an endocytic system protein, was reported to interact and colocalizes with STAT3 in endosomes, regulating pluripotency in mESC and maintaining hematopoietic stem cells in Drosophila.^[^ ^8^ ^]^ In this study, we found in mESC cultured in LIF, STAT3 markedly translocated into multivesicular endosomes (MVEs). MVEs, also known as multivesicular body (MVB), was used to describe intracellular endosomes that contain multiple small vesicles.^[^ ^9^ ^]^ Ultrastructural analysis revealed the presence of MVEs within mESC, characterized by abundant population of intraluminal vesicles (ILVs), which could be secreted as exosomes into the extracellular environment.^[^ ^10^ ^]^

MVEs can either fuse with the lysosomes for degradation or fuse with the plasma membrane to release the ILVs as exosomes.^[^ ^11^ ^]^ What guide MVEs toward the plasma membrane for exosome secretion instead of toward lysosomes for degradation? Previous research has underscored the critical functions of the Rab family of small GTPases alongside SNARE proteins in facilitating exosome secretion.^[^ ^12^, ^13^, ^14^ ^]^ It is noteworthy that subunits of the exocyst have the capability to interact with SNARE proteins or GTPases during the process of exocytosis.^[^ ^15^ ^]^ The exocyst is an octameric protein complex which comprises Sec3, Sec5, Sec6, Sec8, Sec10, Sec15, Exo70, and Exo84.^[^ ^16^ ^]^ A study reported phosphoinositide switch mediates exocyst recruitment to MVEs for exosome secretion.^[^ ^17^ ^]^ Sec15 subunit, also known as Exoc6, has been identified to directly bind with GTP‐bound Rab11, Rab10, and Rab8,^[^ ^18^, ^19^ ^]^ all of which are reported to play a role in the secretion of exosomes.^[^ ^20^, ^21^, ^22^ ^]^ These studies suggest that the exocyst, which is extensively regulated by the Rab family of small GTPases, may play a critical role in the regulation of exosome function.

In the present study, we noted that LIF‐activated STAT3 underwent Sec15b mediated STAT3 MVEs translocation and exosome secretion. Sec15b‐STAT3 exosomes regulate the phosphorylation of T202/Y204‐ERK1/2 and S9‐GSK3β balance to maintained mESC pluripotency. Furthermore, mice with Sec15b knockout exhibit complete embryonic lethality. STAT3 mutant mice (K177R/K180R) were partially embryonic lethal or postnatal‐death, with the living ones were accompanied with development abnormally.

## 2 Results

### 2.1 STAT3 Undergoes Both Nuclear and MVEs Translocation in Stem Cells

**Figure 1:**
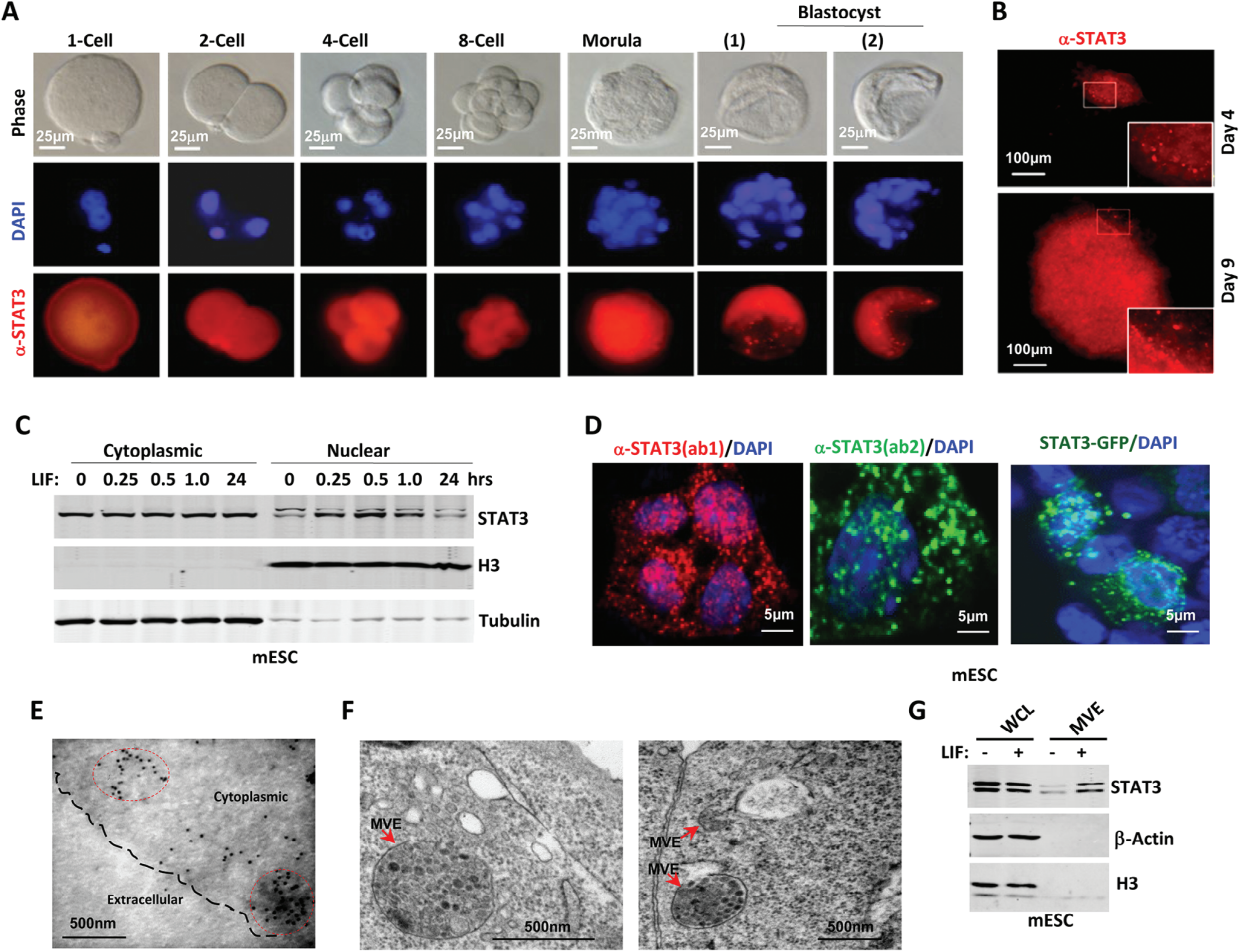
STAT3 undergoes both nuclear and MVEs translocation in mouse embryos and mESC. A) Expression and distribution of STAT3 during preimplantation mouse development. STAT3 was stained with anti‐STAT3 antibody (red), and DNA was stained with 4,6‐diamidino‐2‐phenylindole (DAPI) (blue). B) Immune‐fluorescence staining of endogenous STAT3 in day‐4 and day‐9 outgrowths during mESC derivation. C) E14 cells maintained in 2i were treated with LIF (10 ng mL^−1^) for indicated time and separated for cytoplasmic and nuclear fractions. D) For the mESC maintained in LIF, STAT3, and DAPI were stained and visualized with confocal microscope. E) Immuno‐electron microscope demonstrated the distribution of STAT3. F) MVBs in the mESC were visualized with TEM. G) In 2i maintained E14 cells, MVBs were separated from whole cell lysates and analyzed by Western blotting.

In mouse embryos, we observed STAT3 in cells ranging from the one‐cell stage to the morula stage. Notably, STAT3 began to appear in vesicular structures at the blastocyst stage (**Figure** **1**A), also appear in vesicular structures in the inner cell mass of days 4 and 9 (Figure 1B). Given the STAT3 role as a transcription factor, in mESC maintained in Smith 2i medium, STAT3 rapidly translocated to the nucleus in response to LIF exposure. But nuclear STAT3 levels commenced to diminish in LIF‐containing medium for over an hour (Figure 1C). Unlike the unstable nuclear STAT3, both STAT3 antibody staining and GFP‐STAT3 transfection assays revealed the continuous presence of STAT3 within MVEs in mESC cultured with LIF (Figure 1D; Figure S1A,B, Supporting Information). Consistently, immuno‐TEM analysis confirmed the distribution of STAT3 proteins within the cytoplasm, appearing both as bundles and dispersed spots (Figure 1E). The LIF‐induced vesicular distribution of STAT3 within the cytoplasm did not coincide with markers indicative of mitochondria, lysosomes, autophagosomes, or the Golgi apparatus (Figure S1C, Supporting Information). This LIF‐induced STAT3 vesicular distribution was also observed in the outgrowths from days 6 and 9 during the derivation of mESC (Figure S1D, Supporting Information). Furthermore, the presence of STAT3 in MVEs or in the nucleus was unequivocally detected using an antibody against STAT3 phosphorylated at tyrosine 705 (STAT3‐Y705) in CGR8 cells (Figure S1E, Supporting Information). As STAT3 is critical for the maintenance of inner cell mass lineages,^[^ ^23^ ^]^ and mESC are primarily derived from the inner cell mass, which is located at the blastocyst stage of early embryos. We speculate that STAT3 within vesicles is involved in maintaining the pluripotency of mESC.

In the cytoplasm of stem cells cultured in the presence of LIF, transmission electron microscopy (TEM) revealed the presence of membrane‐enclosed vesicles. These vesicles were observed either as isolated mono‐luminal vesicles or as MVEs, the latter indicated by arrows and containing a high quantity of ILVs (Figure 1F). Cellular cytoplasmic and nuclear fractions were separated using the Cytoplasmic and Nuclear Protein Extraction Kit (Beyotime P0028), with the cytoplasmic fraction subsequently used for isolating intracellular transport vesicles, following the method reported by J. Gruenberg.^[^ ^24^ ^]^ Isolation of MVEs from the cytoplasmic fraction of mESCs revealed that LIF can promote the translocation of STAT3 within MVEs (Figure 1G). These observations suggest that STAT3 may be translocated into MVEs for potential involvement in cellular signaling or maintenance of pluripotency.

### 2.2 The CCD Domain of STAT3 was Responsible for the MVEs Translocation

**Figure 2:**
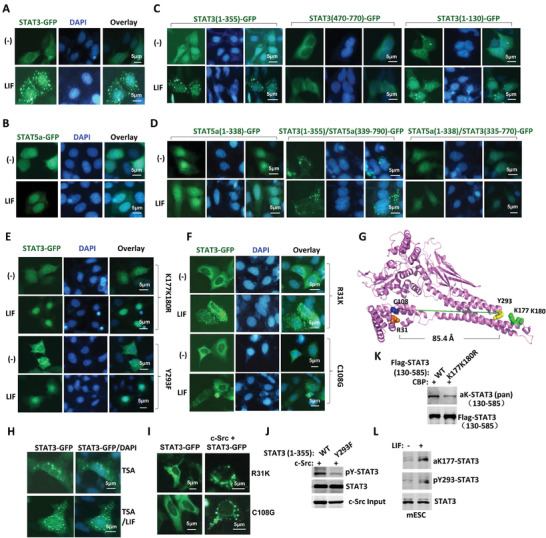
Modifications in the CCD domain are key regulators for STAT3 MVEs translocation. A‐F) HEK293T cells were transiently transfected with STAT3‐GFP (A), STAT5a‐GFP (B), STAT3‐GFP fragments (C), GFP‐tagged STAT3/STAT5 domain swaps (D), GFP‐STAT3 with K177K180R or Y293F mutation (E) or GFP‐STAT3 with R31K or C108G mutation (F) and treated with or without LIF and stained with DAPI. G) The STAT3 protein crystal structure. The residues of R31, C108, K177, K180, and Y293 were indicated in different colors. H) HEK293T cells were transiently transfected with STAT3‐GFP followed by treatment with TSA (100 nM) for 6 h, then the cells were treated with or without LIF and stained with DAPI. I) GFP‐tagged STAT3‐R31K or STAT3‐C108G variant was co‐transfected with or without c‐Src in HEK293T cells. J) Myc‐STAT3 (1‐355) with or without Y293F substitution was co‐transfected with HA‐c‐Src in HEK‐293T cells. Purified STAT3 was analyzed in Western blot for tyrosine phosphorylation (pY20). K) Flag‐STAT3 (130‐585) with or without K177K180R double mutation was co‐transfected with CBP in HEK293T cells. Immunoprecipitated STAT3 was analyzed with pan acetyl‐lysine antibody in Western blot. L) STAT3 was immunoprecipited from CGR8 cells maintained in LIF or LIF withdrawal for 24 h and analyzed in Western blot with poly clonal antibodies prepared for STAT3‐K177 acetylation or ‐Y293 phosphorylation.

In HEK293T cells, LIF induced STAT3 nuclear translocation and vesicle distribution simultaneously (**Figure** **2**A). In contrast, STAT5a responded to LIF treatment solely for nuclear translocation without any vesicle distribution (Figure 2B). To identify the domain of STAT3 responsible for the MVEs translocation reflected as STAT3 vesicle distribution, we constructed GFP‐tagged N‐terminal domain (ND, amino acids 1–130), N‐terminal domain and coiled‐coil domain (ND‐CCD, amino acids 1–355) and the C‐terminal region of STAT3 (CD, amino acids 470–770).^[^ ^25^ ^]^ When HEK293T cells were treated with LIF, STAT3‐(1‐355) translocated to the MVEs and nuclei simultaneously, whereas STAT3‐(1‐130) markedly reduced the MVEs translocation ability without affecting the nuclear translocation ability. In contrast, the STAT3‐(470‐770) failed to respond to LIF (Figure 2C). In addition, STAT5a‐(1‐338) showed nuclear translocation too (Figure 2D).^[^ ^26^ ^]^ Then we swapped STAT3 and STAT5 for their ND‐CCD domain to try to confirm the specific role of the STAT3 ND‐CCD in LIF response. STAT5a swapped with STAT3 ND‐CCD domain became in response to LIF in forming MVEs and nuclear translocation. On the contrary, STAT3 swapped with the ND‐CCD domain of STAT5a failed to respond to LIF for MVEs formation (Figure 2D). These results collectively demonstrated that the both STAT3 and STAT5a contained multiple nuclear localization signal (NLS) motifs in the ND‐CCD domain (1‐355 amino acids for STAT3, 1–338 amino acids for STAT5a), but STAT3 also contained MVEs localization signal in the CCD domain (131‐355 amino acids).

Then we identified the residues of the ND‐CCD domain responsible for STAT3 MVEs translocation and nuclear translocation respectively by constructing a series of point mutation variants (Tables S4 and S5, Supporting Information), with special focusing on acetylated and phosphorylated residues, as these residues were often involved in sub‐cellular organelle translocation. Compared with wild type STAT3, most of the mutants showed no apparent difference in terms of MVEs and nuclear translocations (Figure S2A, Supporting Information), while both STAT3‐K177K180R and STAT3‐Y293F mutants exclusively underwent nuclear translocation without giving cytoplasmic puncta distribution, suggesting their failure to undergo MVEs translocation (Figure 2E). In contrast, STAT3‐R31K and STAT3‐C108G mutants lost nuclear translocation activity without affecting STAT3 puncta distribution pattern (Figure 2F), suggesting R31 and C108 were critical for STAT3 nuclear translocation rather than the MVEs translocation. STAT3 protein crystal structure revealed that while R31 and C108 were clustered on one end of the N‐helical bundle, K177, K180, and Y293 were clustered on another end, with a cutoff distance of 85.4 Å (Figure 2G). In addition to these modifiable residues, several other non‐modifiable residues hold equal importance. For example, STAT3 with D170D171A and E285E286A mutation also failed to undergo puncta distribution without affecting nuclear translocation upon LIF treatment (Figure S2B, Supporting Information). All the results above suggested a critical role of the CCD domain for STAT3 MVEs translocation.

### 2.3 Both Acetylation and Phosphorylation of the CCD Domain Regulate STAT3 MVEs Translocation

The “KXXK” motif in the CCD domain of STAT3 (amino acid 177–180) generally served as a substrate for acetyl‐transferases such as Tip60 and CBP,^[^ ^27^, ^28^ ^]^ and active acetylation and deacetylation processes were expected at this motif. When cells were treated with HDAC inhibitor Trichostatin A (TSA), enhanced STAT3 MVEs translocation was detected (Figure 2H). The non‐receptor tyrosine kinase c‐Src was involved in STAT3 activation in many types of cancer cells.^[^ ^29^ ^]^ To validate whether co‐transfection of c‐Src would enhance STAT3 MVEs translocation bedsides the reported nuclear translocation, STAT3‐R31K and STAT3‐C108G mutants were co‐transfected with c‐Src, and autonomic MVEs translocation emerged without LIF stimulation (Figure 2I). In addition, the specific inhibitor of JAK2 AG490 showed only limited blocking effect on STAT3 MVEs translocation (Figure S2C, Supporting Information), implying that Tyr705 phosphorylation was not a prerequisite for STAT3 MVEs translocation. On the contrary, Nocodazole blocked the STAT3 MVEs translocation, presumably, via its interfering with the polymerization of microtubules in the MVEs formation (Figure S2C, Supporting Information).^[^ ^30^ ^]^

To confirm that the residues Tyr293, Lys177, and Lys780 can be modified by c‐Src or CBP respectively, STAT3‐Y293F mutant was co‐transfected with c‐Src while STAT3‐K177K180R mutant with CBP. In contrast to the wild type, the STAT3‐Y293F mutation significantly reduced c‐Src catalyzed Tyrosine phosphorylation of STAT3 (Figure 2J) while the STAT3‐K177K180R mutation greatly reduced the pan acetylation level of STAT3 (Figure 2K). Mass spectrometry analysis confirmed the acetylation modification of STAT3 at lysine residues 177 (Figure S2D, Supporting Information). We then prepared specific antibodies for Tyr293 phosphorylated and Lyr177 acetylated STAT3 respectively (Figure S2E, Supporting Information). Utilizing these specific antibodies, we clearly detected STAT3 acetylation on Lys177 and phosphorylation on Tyr293 in mESC maintained in the LIF medium but not in medium absence of LIF (Figure 2L). Consistently, we visualized puncta distribution pattern of K177‐acetylated and Y293‐ phosphorylated STAT3 in mESC (Figure S2F, Supporting Information). In short, both CBP/HDAC catalyzed Lysine acetylation and c‐SRC catalyzed Tyrosine phosphorylation of the CCD domain regulated STAT3 MVEs translocation in the mESC.

### 2.4 Sec15b Facilitates STAT3 MVEs Translocation

**Figure 3:**
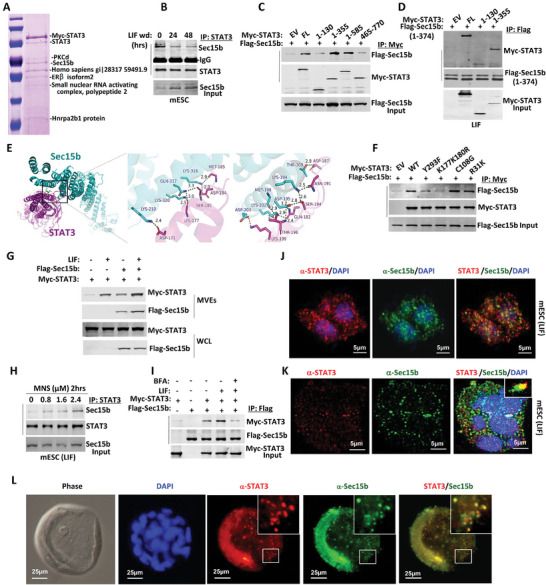
Sec15b facilitates STAT3 MVEs formation. A) Myc‐STAT3 was transiently transfected into cells, STAT3‐pull down proteins were purified by Myc Tag IP Kit and separated in 10% SDS‐PAGE, stained with coomassie brilliant blue followed by in‐gel trypsin digestion of targeted proteins and analysis of the resulting peptide fragments by mass spectrometry. B) Coimmunoprecipitation of STAT3 and Sec15b in the CGR8 mESC upon LIF withdrawal for indicated times. C) Myc‐tagged STAT3 full length (FL) or fragments were transfected with Flag‐Sec15b in HEK293T cells. Anti‐Flag immunoprecipitates were analyzed in Western blot for STAT3 and Sec15b interaction. D) The N‐terminal domains of both STAT3 and Sec15b were transfected into HEK293T cells, protein interaction was confirmed by Western blot. E) Structural analysis reveals a bundle of charged residues of the helical STAT3 N‐terminal domain is involved in the interaction with Sec15b N‐domain. F) STAT3‐Y293F, ‐K177K180R, ‐C108G, or ‐R31K mutants were cotransfected with Flag‐Sec15b in HEK293T cells, anti‐Myc immunoprecipitates were analyzed in Western blot for STAT3 and Sec15b interaction. G) Myc‐STAT3 and Flag‐Sec15b were transfected into HEK293T cells. Transport vesicles from the cytoplasm were isolated and were analyzed in Western blot for STAT3 and Sec15b MVEs translocation. H) The CGR8 mESC was treated with MNS at indicated concentrations for 2 h, protein interaction was checked in Western blot. I) Myc‐STAT3 and Flag‐Sec15b were cotransfected in HEK293T cells, then the cells were treated with BFA as indicated. STAT3 and Sec15b interaction was checked in Western blot. J) Immunostaining of STAT3 and Sec15b in the CGR8 mESC in the presence of LIF. K) Immunostaining of STAT3 and Sec15 in mESC D3 cells. L) Immunostaining of STAT3 and Sec15b in blastocyst on day 3.5.

To elucidate the mechanism behind STAT3 packaging into MVEs, we isolated STAT3‐interacting proteins from LIF‐treated cells and analyzed them using mass spectrometry. Several STAT3‐binding proteins were identified, including the previously reported protein kinase C delta (PKCδ) and estrogen receptor beta (ERβ),^[^ ^31^, ^32^ ^]^ along with the newly discovered Sec15b (**Figure** **3**A). Sec15b is a component of the exocyst complex, which is crucial for the intracellular transport of vesicles and the docking of exocytic vesicles at fusion sites on the plasma membrane.^[^ ^16^ ^]^ In HEK293T cells, Sec15b‐GFP was demonstrated to localize to MVEs, suggesting its involvement in the MVEs translocation process (Figure S3A, Supporting Information).

As expected, STAT3 and Sec15b dissociated from each other in mESC upon LIF withdrawal from the medium and colocalized in cells received LIF treatment (Figure 3B; Figure S3B, Supporting Information). Moreover, Sec15a and Sec15b are highly conserved (80%) members of the Sec15 family. We then investigated the domain of STAT3 responsible for Sec15b interaction. While the shorter STAT3 ND domain (1‐130) was insufficient in Sec15b interaction, the STAT3 ND‐CCD domain (1‐355) was efficient in the association with Sec15b (Figure 3C). Further studies shown that the N‐terminal domain (1‐374) of Sec15b and the N‐terminal domain of STAT3 (1‐355) were involved in a direct “helix‐helix interaction” (Figure 3D). Structural analysis suggested that a cluster of residues in the N‐terminal domain of STAT3 was likely involved in direct interactions with Sec15b during the STAT3‐Sec15b complex formation (Figure 3E; Figure S3C, Supporting Information). Among the mutants tested, the STAT3‐K117K180R and STAT3‐Y293F mutants exhibited significantly weaker interactions with Sec15b, whereas the STAT3‐R31K and STAT3‐C108G variants maintained their ability to interact with Sec15b (Figure 3F). To examine whether the LIF‐induced interaction between Sec15b and STAT3 facilitates the entry of STAT3 into MVEs, we employed the same method described in Figure 1G to isolate transport vesicles from the cytoplasm and assessed STAT3 levels using Western blot analysis. The results demonstrated a significant increase in STAT3 localization within MVEs following LIF stimulation (Figure 3G).

Monensin sodium (MNS) is an ionophore known to interfere with trans‐Golgi protein transport by affecting Golgi membrane Na^+^/H^+^ exchange.^[^ ^33^ ^]^ The interaction between STAT3 and Sec15b reached its peak at 2 h following MNS treatment, exhibiting a dose‐dependent response in mESC (Figure S3D, Supporting Information; Figure 3H). Brefeldin A (BFA), a fungal macrocyclic lactone, acts as a potent inhibitor of vesicle formation.^[^ ^34^ ^]^ Contrary to the effects observed with MNS, pretreatment with BFA disrupted the STAT3‐Sec15b interaction (Figure 3I). Indeed, treatment with MNS (2µM) and BFA (1µM) for 2 h demonstrated opposing effects on the formation of STAT3 MVEs translocation (Figure S3E, Supporting Information). These findings suggest that the Sec15b‐mediated transport of STAT3 to MVEs is subject to precise regulation by protein transmembrane transport. Finally, the interaction between STAT3 and Sec15b was further validated in mESC (Figure 3J). Using N‐storm fluorescence microscopy, we also observed the colocalization of STAT3 and Sec15b within mESC (Figure 3K). Immunofluorescence staining of STAT3 and Sec15b in 3.5‐day mouse blastocysts revealed colocalization of STAT3 and Sec15b within vesicles (Figure 3L). These findings unequivocally demonstrate that STAT3 undergoes Sec15b‐facilitated translocation into MVEs.

### 2.5 The Translocation of STAT3 into MVEs for Secretion as Exosomes

**Figure 4:**
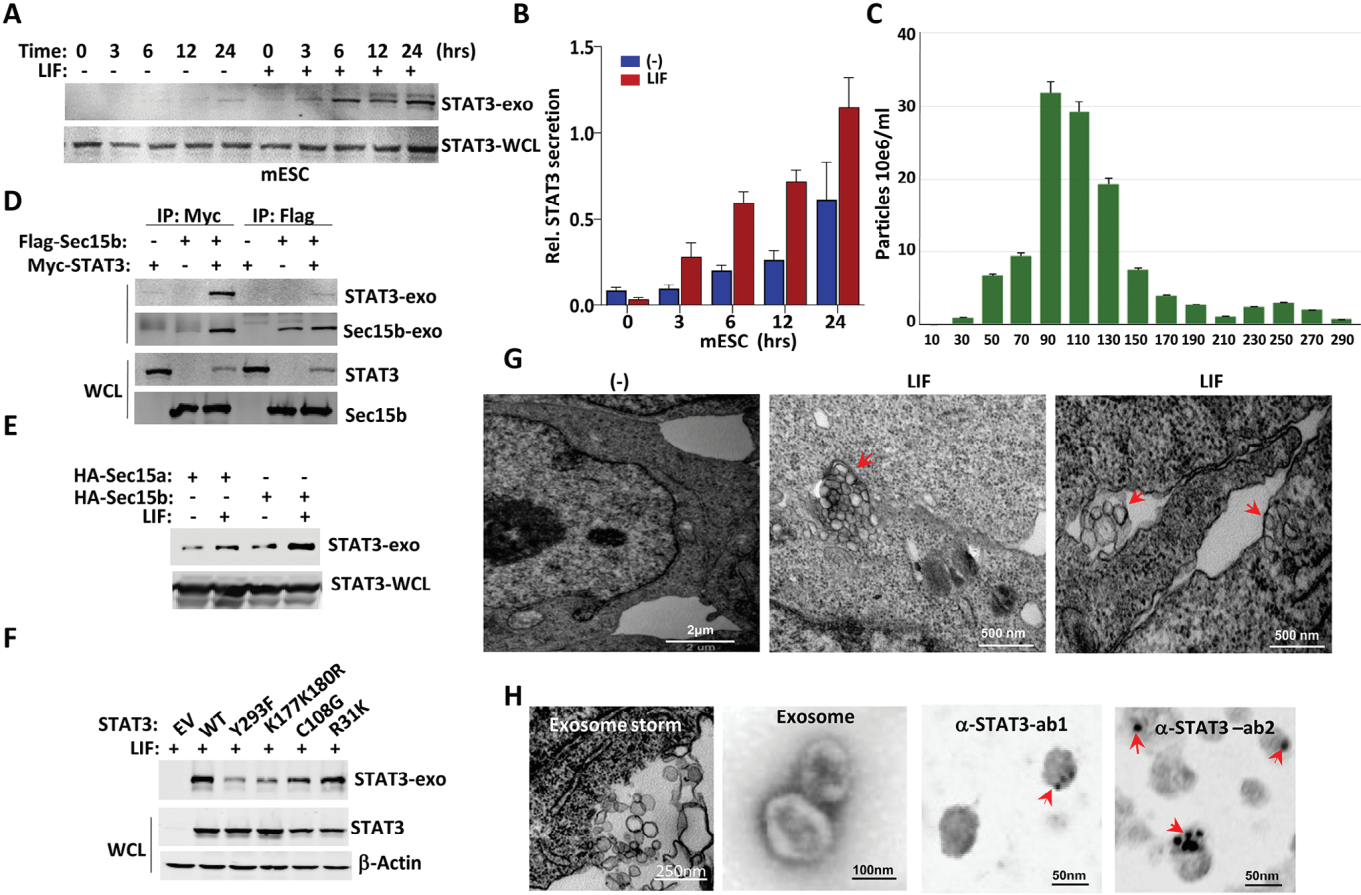
LIF induced Sec15b‐STAT3 secretion. A) The CGR8 mESC was maintained in the presence or absence of LIF for indicated times. STAT3 was blotted from the secreted exosomes collected from the medium or from whole cell lysates. B) The relative amount of STAT3 secretion of (A) was quantitated. Data were shown as mean ± SD, n = 3. C) Diameter of the exosomes obtained from the mESC was determined by the nanosight system. Data were shown as mean ± SD, n = 3. D) Myc‐STAT3 was transiently transfected alone or together with Flag‐Sec15b in HEK293T cells followed by LIF treatment. Secreted exosomes collected from the medium were subjected to coimmunoprecipitation. Both the immunoprecipitates and whole cell lysates were checked in Western blot. E) HA‐Sec15a or HA‐Sec15b was transiently transfected in HEK293T cells followed by LIF treatment or no treatment. Secreted exosomes collected from the medium were subjected to Western blot analysis with STAT3 antibody. F) STAT3‐WT, ‐SY293F, ‐K177K180R, ‐C108G, or ‐R31K was transiently transfected in STAT3^−/−^ MEFs followed by LIF treatment. Secreted exosomes and whole cell lysates were analyzed in Western blot. G) E14 mESC maintained in the LIF‐free medium (left) or LIF‐containing medium (middle and right) were visualized with TEM. In the absence of LIF, the niches between mESC were empty (left). In the presence of LIF, exosomes were visualized either in the niches between two cells (middle) or in the mESC ready for secretion into the niches (right). H) For the CGR8 mESC maintained in LIF, the exosome storm in secretion was visualized (left 1). The morphology of these isolated exosomes was visualized with TEM (left 2). Localization of gold beads with two different antibodies against STAT3 proteins in exosomes was visualized with immuno‐colloidal gold TEM (right 1 and 2). Localization of 10‐nm gold beads in exosomes was indicated (red arrow).

MVEs can fuse with the plasma membrane to release the ILVs as exosomes.^[^ ^11^ ^]^ In CGR8 mESC, LIF induced secretion of STAT3 was observed in a time‐dependent manner, ranging from 3 to 24 h (**Figure** **4**A,B). Exosomes induced by LIF and collected from the mESC typically measured 50–150 nm in diameter and possessed an intact membrane (Figure 4C). STAT3 secretion was significantly increased when STAT3 and Sec15b were co‐transfected into HEK293T cells (Figure 4D), with a similar enhancement observed for LIF stimulation (Figure 4E). As anticipated, the STAT3‐Y293F and STAT3‐K177K180R mutations substantially reduced STAT3 secretion, whereas the STAT3‐C108G and STAT3‐R31K mutations did not affect STAT3 secretion (Figure 4F). After the withdrawal of LIF, MVEs containing exosomes were rarely detected in mESC. In contrast, in mESC cultured in the presence of LIF, MVEs secreting exosomes into the microenvironment or primed for secretion were clearly visualized by using transmission electron microscopy (TEM) (Figure 4G). Finally, the presence of STAT3 in exosomes isolated from the supernatant of mESC cultures was confirmed using immuno‐electron microscopy (immuno‐EM) with two different STAT3 antibodies (Figure 4H).

### 2.6 The Translocation of STAT3 into MVEs Regulates the Balance Between ERK and GSK Signaling Pathways for Maintaining Cellular Pluripotency

**Figure 5:**
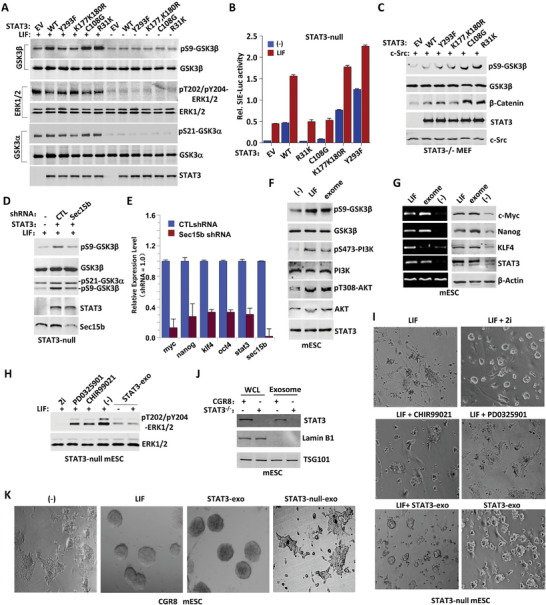
Sec15b‐STAT3 MVEs translocation and secretion maintain cellular pluripotency by modulating the ERK/GSK3β signaling pathway. A) Empty vector or STAT3 variants as indicated was overexpressed in STAT3^−/−^ MEFs followed by LIF (20 ng mL^−1^) treatment for 30 min or not. GSK3α, GSK3β, and ERK phosphorylation were analyzed with indicated antibodies respectively in Western blot. B) The transcriptional activity of STAT3 variants was tested in dual SIE‐luciferase assay. Data were shown as mean ± SD, n = 3. C) Various STAT3 mutants were cotransfected with c‐Src into STAT3^−/−^ MEF cells as indicated, and then western blot was performed by using different antibodies. D) Sec15b was knocked down with specific shRNA (shRNA‐453) in STAT3^−/−^ mESC maintained with LIF. Both GSK3β (S9) and GSK3α (S21) phosphorylation were examined in Western blot. E) Quantitative RT‐PCR analysis of indicated gene expression in LIF‐maintained D3 cells after CTL shRNA or Secl5b specific shRNA was introduced for 72 h. The data were shown as mean + SD. F) The CGR8 mESC, pretreated in LIF withdrawal medium for 24 h, were treated with LIF or STAT3‐exosome (collected from D3 cells) for 30 min. PI3K‐pS473, AKT‐pT308, and GSK3β‐pS9 were estimated in Western blot with indicated antibodies. G) The CGR8 mESC, maintained in LIF‐free medium overnight, were treated with LIF or purified STAT3‐ exosomes for 72 h. RT‐PCR (left) and Western blot (right) were performed to examine indicated gene expression. H) The STAT3^−/−^ mESC were treated with LIF + Smith 2i (3 µM CHIR99021 + 1 µM PD0325901), LIF + CHIR99021, LIF + PD0325901, LIF alone, STAT3 exosome alone (5 mg mL^−1^), or LIF + STAT3‐Sec15 exosome for 48 h. The ERK phosphorylation was analyzed in Western blot. I) The STAT3^−/−^ mESC colonial growth under different conditions: LIF, LIF + Smith 2i, LIF + CHIR99021, LIF + PD0325901, LIF + STAT3‐exosome (5 mg mL^−1^) and STAT3‐exosome. J) Exosomes were isolated from CGR8 mESCs and STAT3^−/−^ mESCs. The expression levels of STAT3, Lamin B1, and TSG101 in exosome and whole cell lysate (WCL) were analyzed by Western blot using the specified antibodies. K) The CGR8 mESC colonial growth under different conditions: LIF withdrawal, LIF (1000 U mL^−1^), STAT3‐exosome (5 mg mL^−1^) obtained from D3 mESC, and exosome (5 mg mL^−1^) obtained from STAT3^−/−^ mESC (upper panel).

Distinct from the wild‐type STAT3, the STAT3‐K177K180R and STAT3‐Y293F mutants, which lacked the capability for translocation into MVEs, displayed diminished GSK3β‐S9 phosphorylation and increased ERK‐T202/Y204 phosphorylation. In contrast, the STAT3 R31K and C108G mutants either did not alter or slightly elevated the level of GSK3β‐S9 phosphorylation (**Figure** **5**A). We then investigated how these two groups of STAT3 mutants regulate their transcriptional activity. STAT3‐K177K180R and STAT3‐Y293F mutants became more active in driving expression of the SIE‐luciferase reporter whereas STAT3‐R31K and STAT3‐C108G mutants lost the transcriptional activity (Figure 5B), suggesting the ability of STAT3 in inducing GSK3β‐S9 phosphorylation and inhibiting ERK activation did not depend on its transcription initiation activity. Since c‐Src greatly favored STAT3‐R31K and STAT3‐C108G mutants in MVEs formation not nuclear translocation (Figure 2I), then we tested the effect of two groups of STAT3 mutants by c‐Src on GSK‐3β S9 phosphorylation. STAT3‐R31K and STAT3‐C108G mutants were more active in GSK‐3β S9 phosphorylation induction as well as in β‐Catenin accumulation (Figure 5C). GSK3β‐S9 phosphorylation can inhibit its own kinase activity and activate the Wnt/β‐catenin pathway by stabilizing β‐catenin, which accumulates and translocates to the nucleus to activate genes critical for cell pluripotency.^[^ ^35^, ^36^ ^]^

Since Sec15b facilitates the STAT3 MVEs translocation and secretion, we knocked down Sec15b in STAT3‐null cells. Our findings showed that LIF‐induced phosphorylation of GSK3β‐S9 requires both STAT3 and Sec15b, as depletion of either abolished this phosphorylation (Figure 5D; Figure S4B, Supporting Information). Besides, Sec15b depletion markedly inhibited expression of the pluripotency associated genes (Figure 5E). As reported, AKT can phosphorylate of GSK3β at Serine 9 and then enhances cellular pluripotency.^[^ ^37^ ^]^ Aligned with these findings, exosomes also stimulate GSK3β phosphorylation at Ser9, at the same time PI3K/AKT pathway activation was detected (Figure 5F). The exosomes also induced the expression of these specific pluripotency genes (Figure 5G) and demonstrated a dose‐dependent effect (Figure S4C, Supporting Information). The activation of the ERK pathway is frequently linked to the suppression of pluripotent gene expression and facilitating endodermal differentiation.^[^ ^38^ ^]^ Exosome also inhibited ERK activation in STAT3^−/−^ mESC, similar to Smith 2i treatment (Figure 5H).

Several studies have also shown that exposing ESCs to exosomes derived from ESCs helps sustain their stem cell properties, even under conditions that usually promote differentiation.^[^ ^39^ ^]^ Extracellular vesicles derived from ESCs can rejuvenate senescent cells and counteract aging in mice.^[^ ^40^ ^]^ Exosomes derived from ESCs also can accelerate wound healing and promote angiogenesis, while significantly reducing the senescence of vascular endothelial cells.^[^ ^41^ ^]^ Thus, the STAT3 MVEs translocation and the subsequent exosome secretion promote the self‐renewal growth in mESCs. We indeed found exosomes promoted clonal growth in STAT3^−/−^ mESC, akin to the effect of Smith 2i (Figure 5I). Additionally, exosomes from wild‐type mES cells (STAT3‐exo), but not those from STAT3‐null mESC (STAT3‐null‐exo), could substitute for LIF in supporting self‐renewal of mESC (Figure 5J,K).

Moreover, these two groups of STAT3 mutants also showed opposite roles on cell cycle regulation, for cells expressing STAT3‐K177K180R or STAT3‐Y293F variant accumulated in G1 or G2/M phase whereas cells expressing STAT3‐C108G or STAT3‐R31K variant accumulated in S phase (Figure S4A, Supporting Information). Taken together, despite STAT3 is a transcription factor that induced gene circuitries involved in pluripotency and formation of early embryonic lineages,^[^ ^3^, ^4^, ^42^ ^]^ the above results strongly indicated that STAT3‐Sec15 exosomes were critical in supporting self‐renewal growth of the mESC via regulating signaling pathways.

### 2.7 STAT3‐K177K180R and STAT3‐R31K Mutant Mice Show Developmental Abnormalities

**Figure 6:**
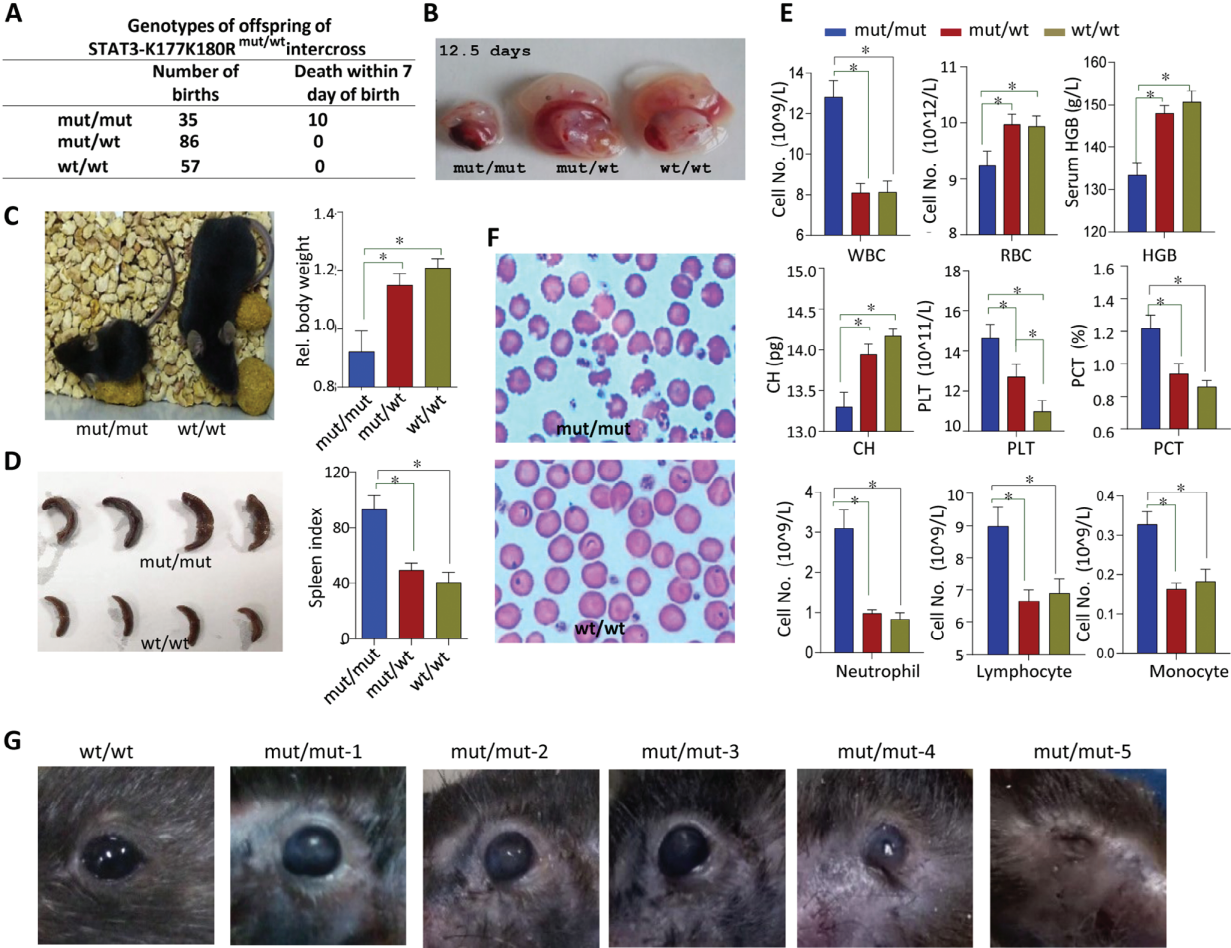
Mice with STAT3‐K177K180R mutant exhibit significant developmental abnormalities. A) The statistical results of offspring genotyping of STAT3‐K177K180R intercross. B) Abnormal phenotypes of STAT3‐K177K180R homozygous (STAT3*^mut/mut^*) embryo on 12.5 days. C) The STAT3*^mut/mut^* mice exhibit slow growth, with both reduced weight and smaller size (letf). The body weight (n = 12) of STAT3 wild type (STAT3*^wt/wt^*), heterozygous (STAT3*^mut/wt^*), or homozygous (STAT3*^mut/mut^*) mice in 12 weeks (right). D) The STAT3*^mut/mut^* mice display splenomegaly (left). The spleen index (n = 6) of STAT3 wild type (STAT3*^wt/wt^*), heterozygous (STAT3*^mut/wt^*), or homozygous (STAT3*^mut/mut^*) mice in 12 weeks (right). E) The number of different categories of peripheral blood cells from STAT3*^mut/mut^*, STAT3*^mut/wt^*, and STAT3*^wt/wt^* mice were analyzed with an automated hematology analyzer (n = 11). F) Red blood cell staining of STAT3*^wt/wt^* and STAT3*^mut/mut^* mice. G) The STAT3*^mut/mut^* mice develop eye abnormalities and even lead to atrophy and blindness in later growth stages.

To gain further insight of STAT3 secretion in embryonic development, we evaluated mice expressing STAT3‐K177K180R mutant (STAT3*^mut/mut^*) generated by the CRISPR/Cas9 gene editing system (Figure S5A, Supporting Information). STAT3*^mut/mut^* mice were 28.5% postnatal death (**Figure** **6**A), and abnormally developed STAT3*^mut/mut^* embryos were found at embryonic days 12.5 (Figure 6B). These results were consistent with the previous findings that STAT3 was essential for early embryonic development as STAT3‐deficient embryos shown a degeneration at embryonic days 7.5.^[^ ^43^ ^]^ The surviving STAT3*^mut/mut^* mice had both reduced body size and weight (Figure 6C) without apparent abnormalities in bone development (Figure S5B,C, Supporting Information). In STAT3*^mut/mut^* mice, we also found abnormal hematopoietic differentiation, characterized by spleen enlargement (Figure 6D), reduced red blood cells (RBC) and hemoglobin (HGB), alongside increased white blood cells (WBC), neutrophil, lymphocyte and monocyte (Figure 6E). RBC morphological abnormalities were detected (Figure 6F). Unexpectedly, notably RBC and HGB counts, along with RBC morphological abnormalities, were observed in mice with a spontaneous exon 8 deletion mutation in the Sec15 gene.^[^ ^44^, ^45^ ^]^ Proper exosome complex levels are essential for maintaining the balance between proliferation and differentiation, critical for RBC development.^[^ ^46^ ^]^ These results suggest that Sec15‐mediated translocation and secretion of STAT3 via exosome play a role in hematopoietic development. Another notable manifestation of developmental abnormalities is that in most homozygous mice, eye damage progressively worsens into the later stages of growth. Initially characterized by a loss of luster and edema‐like symptoms in the eyes, the condition gradually leads to atrophy, eventually resulting in complete blindness (Figure 6G). Additionally, a small number of mice exhibited signs of skin damage (Figure S5D, Supporting Information).

We also generated STAT3‐R31 mutant mice and found that the STAT3‐R31 mutation resulted in embryonic lethality, highlighting the crucial role of STAT3's nuclear translocation in regulating gene transcription (Figure S7A,B, Supporting Information). Recent research showed that exosomes containing STAT3 can promote resistance to 5‐FU in colorectal cancer cells. Upon introduction of these exosomes, a significant accumulation of phosphorylated STAT3 (p‐STAT3) was detected in the nuclei of the recipient cells.^[^ ^47^ ^]^ This suggests that STAT3, once secreted in exosomal form, may reenter the nuclei of other cells and continue to regulate gene transcription.

### 2.8 Gene Expression is Abnormal in the Embryos of STAT3*^mut/mut^* Mice and Embryonic Lethality in Sec15b^−/−^ Mice

**Figure 7:**
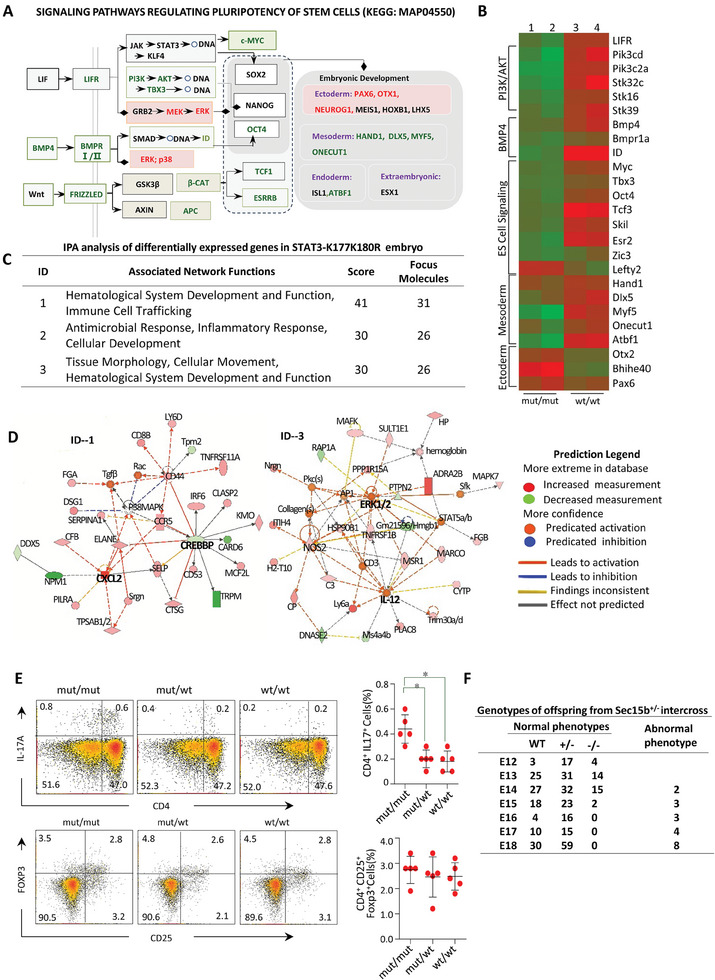
STAT3‐K177K180R mutation in mice exhibit reduced embryonic stem cell pluripotency and hematopoietic abnormalities. A) Analysis of differentially expressed genes regulating pluripotency in STAT3*^mut/mut^* mouse embryos. Downregulated genes are shown in green, and upregulated genes are displayed in red. B) Heat map of related gene expression. Most of genes involved in regulating pluripotency of mouse stem cells and mesoderm development were down‐regulated in STAT3*^mut/mut^* embryos. In contrast, some genes involved in MAPK pathway and ectoderm development were up‐regulated. C) Ingenuity Pathway Analysis (IPA)‐identified top 3 most significant gene networks with score ≥30. D) IPA network analysis of differentially expressed genes related hematological system development. E) In vivo differentiation of Th17 cells and Treg cells from STAT3*^mut/mut^*, STAT3*^mut/wt^*, and STAT3*^wt/wt^* mice were detected with FACS. F) The statistical results of offspring genotypes from Sec15b^+/−^ intercross.

To further assess the impact of the STAT3‐K177/180R mutation on gene expression during embryonic development, embryos at 15 days of gestation from the same uterus were subjected to RNA sequencing. Compared with only 37 genes up‐regulated and 60 genes down‐regulated in 30 478 genes assayed between STAT3*^wt/mut^* and STAT3*^wt/wt^* mice, gene expression in STAT3*^mut/mut^* mice changed significantly with 2126 genes upregulated and 2913 genes downregulated in STAT3*^mut/mut^* embryos (Figure S5E, Supporting Information). Further analysis, based on embryonic sequencing results, focused on genes associated with mouse stem cell pluripotency (KEGG: MAP04550). The result revealed a downregulation of several pluripotency‐related genes (c‐Myc, Tbx3, Oct4, Esrrb) in the embryos of mutant mice (**Figure** **7**A,B). Research indicates that the ectoderm gives rise to the skin and nervous system, while the hematopoietic system primarily develops from the mesoderm.^[^ ^48^ ^]^ The abnormalities in ectoderm and mesoderm gene expression are consistent with the developmental anomalies observed in STAT3*^mut/mut^* mice (Figure 6).

Subsequent Ingenuity Pathway Analysis (IPA) of differentially expressed genes identified the top three gene networks and their related functions. Notably, abnormal embryonic development, particularly of the hematological system, was enriched in STAT3*^mut/mut^* embryos (Figure 7C). Central nodes impacting hematological system development and function were identified as ERK1/2 and the CREB‐binding protein (also known as CREBBP or CBP) (Figure 7D). The upregulation of the ERK1/2 node consistent with findings that the STAT3 K177R/K180R mutant attenuates inhibition on ERK1/2‐T202/Y204 phosphorylation (Figure 5A). CBP shares very high‐sequence similarity with another acetyltransferase p300, we reported p300 mediated STAT3 acetylation on K‐685.^[^ ^49^ ^]^ In this study, we found CBP mediated STAT3 acetylation on K177‐K180 (Figure 2K). The activation of the ERK pathway serve as a stimulus for HSCs to exit the self‐renewal program and transition into the differentiation phase.^[^ ^50^ ^]^ CREBBP activity in bone marrow stromal cells influences the microenvironment that supports HSCs, that loss of CREBBP in murine haematopoietic stem and progenitor cells (HSPCs) leads to increased development of B‐cell lymphomas.^[^ ^51^ ^]^ Previous studies have shown that CREB is a classical target of PI3K/AKT signaling.^[^ ^52^ ^]^ In our study, adding exosomes to embryonic stem cells significantly upregulated PI3K and AKT phosphorylation (Figure 5E). The STAT3‐K177/180R mutation leaded to reduced STAT3‐exosome levels, which consistent with the downregulation of the CREBBP target in the embryos of STAT3‐K177/180R mutant mice. CBP knockdown also enhances signaling of the MEK/ERK pathway in acute lymphoblastic leukemia,^[^ ^53^ ^]^ which is also consistent with the up‐regulated ERK1/2 in STAT3*^mut/mut^* embryos. Alterations in the ERK and CREBBP signaling pathways in embryos imply enhanced hematopoietic stem cell differentiation. In line with this, we observed a significant increase of myeloid and lymphoid cells in the peripheral blood of STAT3*^mut/mut^* mice.

We observed that the interactions between STAT3‐K177/180 and STAT3‐C108 modulate the balance of the ERK and GSK3β signaling pathways, functioning similarly to a seesaw (Figure 5A). Previous studies suggest that modifications at the STAT3‐C108 site are intimately linked to Th17 cell differentiation.^[^ ^54^ ^]^ This raises the question: Does a mutation at the STAT3‐K177/180 sites inversely enhance the functionality at the STAT3‐C108 site? Flow cytometry analyses revealed an increased proportion of Th17 cells in the peripheral blood of mice harboring mutations at the STAT3‐K177/180 sites (Figure 7E). These findings underscore the vital role of STAT3 translocation into MVEs in preserving the equilibrium of intracellular signaling.

To confirm the role of Sec15b in embryonic development, we also prepared Sec15b^loxp/loxp^ mouse by inserting Loxp into the upstream and downstream of exon2 of Sec15b gene (Figure S6A,B, Supporting Information). However, after crossing with CMV‐cre mice, no Sec15b^−/−^ offspring were found. Sec15b^−/−^ homozygous embryos with normal phenotype in the uterus was only obtained within 16 days after pregnancy, suggesting that Sec15b^−/−^ mice were embryonic lethal (Figure 7F).

## 3 Discussion

Although the majority of studies have focused on STAT3's Y705 phosphorylation and its role as a transcription factor in regulating gene transcription within the nucleus, over 80 amino acids in STAT3 subject to post‐translational modifications have been identified in advancing research, such as phosphorylation, acetylation, methylation, ubiquitination, and even palmitoylation.^[^ ^55^ ^]^ Post‐translational modifications confer upon STAT3 the ability to localize to various cellular compartments, including the nucleus, mitochondria, endosomes, and plasma membrane, thereby facilitating its range of functions.

In this study, we discovered that acetylation at STAT3's K177/K180 sites and phosphorylation at the Y293 site facilitates its entry into MVEs, while the C108 and R31 sites are closely associated with STAT3's nuclear localization. STAT3 is palmitoylated at C108, which enhances its recruitment to membranes and promotes Th17 cell differentiation has been reported.^[^ ^54^ ^]^ Additionally, arginine methylation of STAT3 by PRMT1 facilitates astrocyte differentiation, with R31 identified as a potentially crucial site.^[^ ^56^ ^]^ A study revealed that EZH2 methylates STAT3 at the K180 site, promoting clonogenic growth in glioblastoma multiforme, characterized by a subpopulation of stem‐like cells.^[^ ^57^ ^]^ Here, we found that STAT3's K177/K180 and Y293 sites are involved in MVEs translocation for maintaining the pluripotency of mESC.

Recently, Wei Guo et al found Sec15b partially colocalized with MVEs marker CD63.^[^ ^17^ ^]^ STAT3‐K177K180R inhibited sec15b facilitates STAT3 MVEs translocation and subsequent secretion. STAT3‐K177K180R mutant (STAT3*^mut/mut^*) mice were generated to further elucidate the role of STAT3 translocation to MVEs in embryonic development. Surviving STAT3*^mut/mut^* mice demonstrate significant developmental abnormalities, especially the hematopoietic system, in later growth stages. Defects in hematopoietic differentiation, including notably reduced counts of RBCs and HGB, as well as morphological abnormalities in RBCs, were also observed in mice harboring a spontaneous deletion mutation in exon 8 of the Sec15 gene.^[^ ^44^, ^45^ ^]^ Developmental anomalies observed in STAT3*^mut/mut^* mice also share partial similarities with the clinical symptoms of STAT3 gain‐of‐function (GOF) mutations, such as elevated leukocyte levels and stunted growth. Within STAT3 GOF syndrome, 73% of patients exhibited lymphoproliferation, primarily presenting as diffuse lymphadenopathy and/or splenomegaly. Additionally, growth failure was a prevalent symptom, affecting 57% of affected individuals.^[^ ^58^ ^]^ Blood development starts with self‐renewing of HSCs.^[^ ^59^ ^]^ Here, a series of abnormalities were observed in the blood system of STAT3*^mut/mut^* mice, this may be related to the destruction of HSC self‐renewal growth and differentiation.

LIF activation commonly triggers the PI3K, ERK, and STAT3 signaling pathways in cells.^[^ ^5^ ^]^ ERK pathway activation often leads to the downregulation of pluripotency genes and actives differentiation.^[^ ^60^ ^]^ PI3K/Akt signaling promotes cellular pluripotency by phosphorylating GSK3β at Serine 9.^[^ ^61^ ^]^ GSK3β‐S9 phosphorylation triggers β‐catenin stabilization and Esrrb activation, essential for the self‐renewal in mESC.^[^ ^62^, ^63^ ^]^ STAT3 mutants (K177K180R and Y293F), unable to enter MVEs, exhibited reduced GSK3β‐S9 phosphorylation and enhanced ERK‐T202/Y204 phosphorylation. STAT3‐R31K and STAT3‐C108G mutants were more active in GSK‐3β S9 phosphorylation induction as well as in β‐Catenin accumulation. Embryonic sequencing results of STAT3*^mut/mut^* mice also revealed an upregulation of the ERK pathway and a downregulation of the CREBBP signaling pathway. Activation of the ERK pathway serves as a stimulus for HSCs to exit the self‐renewal program and transition into the differentiation phase.^[^ ^50^ ^]^ The loss of CREBBP in murine HSPCs results in an increased development of B‐cell lymphomas.^[^ ^51^ ^]^ Indeed, in STAT3*^mut/mut^* mice, we observed a marked increase in both myeloid and lymphoid cells in peripheral blood. These findings highlight the essential role of Sec15b‐mediated STAT3 transport to MVEs in maintaining cellular pluripotency, with disruptions in this process leading directly to developmental abnormalities in the hematopoietic system.

We generated Sec15b‐null (Sec15b^−/−^) mutant mice and found that these mice were embryonically lethal. This underscores the critical importance of Sec15b in sorting proteins and subsequently transporting them to MVEs. STAT3‐R31K mutant mice were embryonically lethal too. This highlights the critical role of STAT3 in nuclear gene transcription. STAT3, after being secreted via exosomes, can re‐enter recipient cell nuclei to regulate transcription.^[^ ^47^ ^]^ Thus, how secreted STAT3 influences recipient cell function merits further investigation.

In summary, our study demonstrates that the interaction between STAT3 and Sec15b facilitates the translocation of STAT3 to MVEs and its subsequent secretion. This translocation regulates the intracellular balance of the ERK and GSK3β pathways, thereby playing a crucial role in maintaining cellular pluripotency. The absence of this mechanism leads to aberrant embryonic development, particularly affecting the development of the hematopoietic system.

## 4 Experimental Section

**Key Resources Table**

**Table:** 

REAGENT	SOURCE	IDENTIFIER
Antibodies		
STAT3 F‐2	Santa Cruz Biotech	Cat# sc‐8019; RRID: AB_628293
STAT3 C‐20	Santa Cruz Biotech	Cat#sc‐482; RRID: AB_632440
pY705‐STAT3	Santa Cruz Biotech	Cat# sc‐7993; RRID: AB_656682
c‐Myc9E10	Santa Cruz Biotech	Cat#sc‐40; RRID: AB_627268
Oct‐3/4 C‐10	Santa Cruz Biotech	Cat# sc‐5279; RRID: AB_628051
GKLF	Santa Cruz Biotech	Cat# sc‐20691; RRID: AB_669567
pY20	Santa Cruz Biotech	Cat# sc‐508; RRID: AB_628122
Ac‐Lys (AK‐4)	Santa Cruz Biotech	Cat# sc‐32852; RRID: AB_634874

**Table:** 

REAGENT	SOURCE	IDENTIFIER
pY467‐PI3‐kinase p85α	Santa Cruz Biotech	Cat#sc‐293115; RRID: AB_10844180
PI3‐kinase p85α	Santa Cruz Biotech	Cat# sc‐423; RRID: AB_632211
pT308‐AKT1	Santa Cruz Biotech	Cat# sc‐135650; RID: AB_2224730
AKT1	Santa Cruz Biotech	Cat# sc‐5298; RRID: AB_626658
Myc Tag	Santa Cruz Biotech	Cat# sc‐789; RRID: AB_631274
pY279/Y216‐GSK3α/β	Santa Cruz Biotech	Cat#sc‐81496; RRID: AB_1125866
STAT3	Cell Signaling Tech	Cat#9139; RRID: AB_331757
pT202/Y204‐ERK	Cell Signaling Tech	Cat#4370; RRID: AB_2315112
ERK	Cell Signaling Tech	Cat#4695; RRID: AB_390779
pS21/S9‐GSK3α/β	Cell Signaling Tech	Cat#9327; RRID: AB_659961
GSK3α	Cell Signaling Tech	Cat#9338; RRID: AB_2114897
Phospho‐GSK‐3β (Ser9)	Cell Signaling Tech	Cat# 9336, RRID: AB_331405
GSK‐3β (D5C5Z)	Cell Signaling Tech	Cat# 12456, RRID: AB_2636978
Actin	Beyotime	Cat#AA128; RRID: AB_2861213
c‐Myc	Bioss	Cat#bs‐0334R; RRID: AB_10855782
Nanog	Bioss	Cat#bs‐0829R; RRID: AB_10857750
Flag M2	Sigma‐Aldrich	Cat#F1804; RRID: AB_262044
GFP	Roche	Cat#11814460001; RRID: AB_390913
Sec15b	Abcam,	Cat# ab105075; RRID: AB_10712475
TSG101 (E6V1X)	Cell Signaling Tech	Cat# 72312; RRID: AB_2927716
Lamin B1 (119D5‐F1)	Santa Cruz Biotech	Cat# sc‐56143; RRID: AB_2136302

**Table:** 

REAGENT	SOURCE	IDENTIFIER
Goat anti‐rabbit RDye® 680	LI‐COR Biosciences	Cat#926‐32221; RRID: AB_621841
Goat anti‐mouse RDye ® 800CW	LI‐COR Biosciences	Cat#926‐32210; RRID: AB_621842
Goat anti‐rabbit IgG Chromeo 546	Abcam	Cat#ab60317; RRID: AB_954976
Goat anti‐rabbit IgG Chromeo 488	Abcam	Cat#ab60314; RRID: AB_954969
Alexa Flour 555 donkey anti‐mouse	Invitrogen	Cat#A31570; RRID: AB_2536180
Chemicals, peptides, and recombinant proteins		
GMEM	Gibco	Cat#11710035
DMEM	Gibco	Cat#11965092
N2	Gibco	Cat#17502001
B27	Gibco	Cat#17504044
DAPI	Sigma	Cat#D9542
CHIR99021	Qcbio Science & Technologies	Cat#04‐0004
PD0325901	Qcbio Science & Technologies	Cat#04‐0006
AG490	Beyotime	Cat# S1509
Trichostatin A	Beyotime	Cat# S1893
Nocodazole	Beyotime	Cat# S1765
LY294002	Beyotime	Cat# S1737
Monensin	Beyotime	Cat# S1753
Nicotinamide	Beyotime	Cat# S1761
5‐(N,N‐Dimethy)amiloride hydrochloride (DMA)	Sigma	Cat# A4562
ESGRO® mLIF	Millipore	Cat# ESG1107
recombinant human LIF	Millipore	Cat# LIF1005
recombinant mouse LIF	Millipore	Cat# LIF2010
Critical commercial assays		
JC‐1 kit	Beyotime	Cat# C2006
Lyso‐Tracker Red	Beyotime	Cat# C1046
Nuclear protein extraction	Beyotime	Cat#P0028
c‐Myc Tag IP/Co‐IP kit	Thermo Scientific	Cat#23625
QuikChange® XL Site‐directed mutagenesis kit	Stratagene	Cat#200517
Cycle TESTTM PLUS kit	Becton Dickinson	Cat#340242
Prime Script kit	Takara	Cat#RR014A
Wright's staining solution	Nanjing Jiancheng Bioengineering Institute	Cat#D007
Mouse Regulatory T Cell Staining Kit	MultiSciences Biotech Co., Ltd	Cat#KTR201
Mouse Th17 Staining Kit	MultiSciences Biotech Co., Ltd	Cat#KTH217
Deposited data		
The mice embryo of STAT3 K177/180R RNA‐seq	This paper	GSE271656

**Table:** 

REAGENT	SOURCE	IDENTIFIER
Experimental models: Organisms/strains		
Mouse: STAT3‐k177/180R mutant	This paper	Shanghai Model Organisms
Mouse: Sec15b‐knockout	This paper	Shanghai Model Organisms
Mouse: STAT3‐R31K mutant	This paper	Shanghai Model Organisms

### Mice, Embryo, and Embryonic Outgrowth

Mouse blastocysts collected 3.5days post coitum were flushed from the uteri of the B6D2F1 (C57BL/6xDBA2) female mice containing copulatory plugs. Alternatively, about 21hr post human chorionic gonadotropin injection, zygotes were obtained by incubation with hyaluronidase followed by several washes in KSOM (Millipore) drops. The embryos were then cultured in KSOM at 37°C under 5% CO2 in air to collect the embryos. The blastocysts were harvested and the zonapellucida was removed using acid Tyrode's solution. Each blastocyst was transferred into one well of a 96‐well plate seeded with irradiated feeder cells in Knockout DMEM supplemented with 20% Knockout^TM^ serum replacement (Gibco) and LIF (2000 U mL^−1^) for 4, 6, and 9 days. This animal experiment was performed according to the ‘Regulations for the Care and Use of Laboratory Animals in Zhejiang Chinese Medical University’. The Institutional Animal Care and Use Committee of Zhejiang Chinese Medical University (SYXK 2013‐0184) approved the study protocol, the certificate number of ethical approve of research procedures is ZSLL‐2017‐177.

### mESC Culture

CGR8 cells were cultured feeder‐free in Glasgow's minimum essential medium (GMEM) using gelatinized flasks while D3 cells were cultured on feeder cells in DMEM‐high glucose medium. STAT3^−/−^ mESC were cultured on feeder cells in N2/B27 medium with 2i, PD0325901 (1µM) and CHIR99021 (3µM). All mESC were maintained in the medium supplemented with 15% fetal bovine serum (FBS) (Gibco) and 1000 U mL^−1^ of LIF.

### mESC, Embryo, and Outgrowths Immunofluorescence Staining

mESC line, mouse ESC outgrowths, or mouse pre‐implantation embryos (with the zona pellucid removed by using acid Tyrode's solution) were washed once with PBS or PBST upon medium removal, fixed with 4% paraformaldehyde for 15 min and permeated with 0.2% tritonX‐100 for additional 15 min followed by three PBS or PBST washes. The cells or embryos were then incubated with anti‐STAT3 (Cell Signaling, 1:100‐250, or Santa Cruz 1:100) or anti‐Sec15b (1:100‐150) at 4°C overnight followed by PBS or PBST rinsing and the secondary antibody incubation for 1 h at room temperature. After extensive PBS or PBST washes, the immunofluorescence staining cells were stained with DAPI.

### Mitochondria, Lysosome Staining

STAT3‐GFP was transiently transfected in HEK‐293T cells. After LIF treatment for 30 min, the HEK‐293T cells were stained with different subcellular organelle biomarkers by using commercial kits including mitochondrial assay kit JC‐1 (Beyotime C2006), Lyso‐tracker red (Beyotime C1046), and visualized with confocal microscopy.

### Transmission Electron Microscope (TEM) and Immuno‐TEM Analysis

CGR8 cells, cultured in LIF or LIF withdrawal for 72 h, were collected, fixed, and embedded for TEM analysis by using HITACHI‐H‐7650 electron microscope. In brief, the cells were fixed with 2.5% glutaraldehyde in PBS (0.1 M, pH7.0) for > 4 h, washed with PBS 3 times (0.1M, pH7.0), and post‐fixed with 1% OsO_4_ in PBS (0.1M, pH7.0) followed by another 3 times PBS washes. After double fixation, the cells were dehydrated with graded ethanol series and incubated with acetone for 20 min. The cells were placed in 1:1 mixture of acetone and the final Spurr resin mixture for 1 h at room temperature followed by transferring to 1:3 mixture of acetone and the final resin mixture for 3 h and to final Spurr resin mixture for overnight. Embedding and ultrathin‐sectioning were done by placing the cells in an Eppendorf tube containing embedding medium for heating at 70°C for 9 h. The cell specimen sections were stained by uranyl acetate and alkaline lead citrate for 15 min respectively and submitted to TEM visualization.

For exosome immuno‐TEM analysis, exosome prepared from mESC (20 mL) was mixed with the primary AB (1:50 dilution with 1% BSA, 20 µL), loaded onto copper mesh, and incubated for 2 h at room temperature. After 3 times PBS washes, 10 nm gold‐labeled secondary AB (Sigma) was added to incubate for additional 30 min at room temperature followed by 4 times PBS washes.Such prepared exosomes were stained with uranyl acetate for 30 s and subjected to TEM analysis.

### Preparation of STAT3 Binding Proteins for Mass Spectrometry Analysis

mESC or HEK‐293T cells were transiently transfect with Myc‐tagged STAT3 by using SuperFectin^TM^ II DNA transfect reagent (PuFei 2012–100) for 36–48 h. The transfection cells were re‐suspended in lysis buffer with the cocktail of protease inhibitors added and incubated on ice for 30 min. Myc‐STAT3 and Myc‐STAT3 binding proteins were immune precipitated from the lysate proteins (100 µg) by using Pierce Mammalian c‐Myc Tag IP/Co‐IP kit following the protocol (Thermo Scientific, 23625). Purified Myc‐STAT3 and Myc‐STAT3 binding proteins were separated in 10%SDS‐PAGE, stained with Coomassie brilliant blue, and submitted for in‐gel trypsin digestion of unique bands prior to ion‐trap tandem mass spectrometry (ms/ms) identification.

### STAT3 and Sec15b Gene Sub‐Cloning and STAT3 Site‐Directed Mutagenesis

Primers used for sub‐cloning of STAT3 and STAT5a (full length, domains, or domain‐swabbed forms) were given in Table S1 (Supporting Information). pEGFP‐C, pcDNA3.1‐2xFlag and pcDNA3.1‐6xMyc were used to construct GFP‐, Flag‐, or Myc‐tagged forms of STAT3, STAT5a, and Sec15b. Primers used for sub‐cloning were given in Table S2 (Supporting Information). Site‐directed mutagenesis was performed following the instruction of the QuikChange® XL Site‐directed mutagenesis kit (Stratagene, La Jolla, CA) and all the primers used for site‐directed mutagenesis of above genes were shown in Table S4 (Supporting Information).

### Endosome, Cytoplasmic and Nuclear Fractions, Co‐Immunoprecipitation, and Western Blot

Cytoplasmic and nuclear fractions were separated with nuclear and cytoplasmic protein extraction kit (Beyotime P0028) and endosomes were then prepared from the cytoplasmic fraction as described previously.^[^ ^24^ ^]^ Briefly, the post‐nuclear supernatant (PNS) was brought to 40.6% sucrose and 0.5 mM EDTA, loaded at the bottom of an SW 60 tube, sequentially overlaid with 1.5 mL 16% sucrose in D_2_O, 3 mM imidazole pH 7.4 containing 0.5 mM EDTA, then 1.0 ml 10% sucrose in D_2_O, 3 mM imidazole pH 7.4 containing 0.5mM EDTA and finally 0.5 ml homogenization buffer (250 mM sucrose, 3mM imidazole pH 7.4). The gradient was run for 60 min at 35 000 rpm using an SW 60 rotor. Endosomes were collected at the 16‐10% sucrose interface. For co‐immunoprecipitation and Western blot analysis: the cell lysates prepared with lysis buffer (0.5–1 mg) were incubated with 1 µg of protein A/G‐agarose conjugated AB for 6 h up to overnight, 4°C. After three lysis buffer washes, the immune precipitates proteins were denatured by boiling in 2x loading buffer for 5 min, separated in SDS‐PAGE, and analyzed with specific antibodies in Western blot.

### Antibody Preparation

Polyclonal ABs against aK177‐STAT3 and pY293‐STAT3 were prepared by immunizing rabbits with synthetic peptides (Ab‐Land Bio‐Tech, Hangzhou, ZJ, China). The peptides corresponding to the sequence region unique to aK177 of STAT3 (CLQDDFDFNY[K]aTLKS) and pY293 (KVS[Y]pKGDPIVQHRPC) of STAT3 were synthesized by Gil Biotech Shanghai. The titer and specificity of these two antibodies were examined with both designed ELISA as well as slot blot analysis by immobilizing the peptides with modification versus control peptides (without modification). Antibody purification was performed by applying modified‐peptide affinity column and unmodified‐peptide cross‐adsorption.

### Exosome Preparation

Fifty milliliter conditioned medium collected from mESC (CGR8, D3, or STAT3^−/−^) in 5×10 cm flasks with confluent growth were pooled together. The medium was then subjected to two successive centrifugations (1000g for 30 min and 10 000g for 1 h). The supernatant was filtered through a 0.45 micron membrane and the exosome pellets were collected upon ultracentrifugation for 2 h at 100,000g.^[^ ^64^ ^]^ Alternatively, the supernatant was filtered through a 0.45micron membrane and was incubated with 1 µg of anti‐STAT3 or anti‐Sec15b for overnight at 4°C followed by incubating with protein A/G‐agarose beads for additional 4 h and washed with lysis buffer. Exosome protein components were analyzed with Western blot or immuno‐TEM. For exosome activity analysis including mESC colonial growth, intracellular signaling pathway activation, and gene regulation, 5 mg mL^−1^ exosome was routinely used.

### Luciferase Reporter Assay and Cell Cycle Analysis

STAT3^−/−^MEFs, in 24‐well were transiently transfected with the corresponding cDNA using Xfect^TM^Polymer. The luciferase reporter (2xIRF1‐SIE‐luciferase) activity was assayed with the dual‐Luciferase reporter assay system (Promega, E1910) by following the manufacturer's instructions. All the samples were tested in triplicates, and the results were normalized relative topRL‐SV40 activity. STAT3^−/−^ MEFs were transfected with STAT3 and variants using Xfect^TM^Polymer reagent and the cell cycles were detected by using flow cytometry according to the protocol of Cycle TESTTM PLUS kit (Becton Dickinson, 340242).

### Quantitative RT‐ PCR

Total RNA was extracted by Trizol reagent. Reverse transcription (RT)‐PCR was performed using Prime Script kit (Takara). All the primers used for pluripotent state gene amplification were shown in Table S3 (Supporting Information). All samples were tested in triplicates, and the results were normalized relative to Actin expression as mean ± SD.

### Sec15b Down Regulation in CGR8 Cells

Two interfering Sec15b gene sequences were constructed into lentiviral expression construct, i.e., LV3‐GFP‐ShSec15b‐453(GCTGAAGCAGTGTCGACTACTACA) and LV3‐GFP‐ shSec15b‐1020 (GTGCCCGTG AAACATTTGA). The LV3‐GFP‐ShSec15b plasmid and the packing plasmids (pGag/Pol, pRev, and pVSV‐G) were mixed proportionately and transfected into HEK‐293T cells using Lipofectamine2000. Six hours after transfection, the medium was replaced with fresh one for additional 72 h. Cell culture medium containing virus was subjected to centrifugation (4000×rpm for 5 min). The supernatant was filtered through a 0.45 µm membrane and the lentivirus pellet was collected upon ultracentrifugation (20 000× rpm for 2 h). HEK‐293T cell was used to detected viral titers. To test Sec15b down regulation, the above prepared lentivirus (80 µL) was incubated with CGR8 cells in 6‐well plate for 24 h. The medium was replaced with the fresh one for CGR8 cells to grow for additional 72 h. Fluorescence microscope was used to measure the proportion of GFP‐positive lentivirus to determine the lentiviral vectors packaging efficiency. Sec15b silencing efficiency in the cells was analyzed with RT‐PCR or Western‐Blot.

### Generation of Sec15b‐Deficient Mouse

The BAC clone containing the Sec15b gene (bMQ250m11, Source Bioscience) was identified in the mouse BAC (bMQ) Library. A loxP site was inserted downstream of exon 2, and the FRT‐neo‐FRT‐loxP cassette was inserted upstream of exon 2 in the targeting vector (Figure S6A, Supporting Information). Exon 2 can be deleted by the Cre‐loxP system. The deletion of the Exon2 will prevent the translation of Sec15b mRNA and cause the loss of the Sec15b protein. The linearized targeting vector was electroporated into ES cells, and neo‐resistant ES cell clones were selected by G418 (300 mg L^−1^) and Ganciclovir (2 µmol L^−1^). The positive ES cell clones of homologous recombination were screened by PCR. A forward primer of Exoc6b‐5p111 (5'‐GGTCTAGTGGGTCTC TTAACTCATCCTGT‐3') and a reverse primer Neo‐Rh (5'‐GGCCTACCCGCTT CCATTGCTC‐3') were used to amplify for 5 ‘homologous recombination arm. The clones with a 3.52‐kb band were the targeted ES cells. A forward primer of Exoc6b3p211 (5’‐CAAAGATGCTGTGAACACAAA ATAATGAG‐3') and a reverse primer Neo‐Lh (5'‐CCGTGCCTTCCTTGA CCCTGG‐3') were used to amplify for 3 ‘ homologous recombination arm. The clones with a 3.52‐kb band were the targeted ES cells. The targeted ES cells were microinjected into C57BL/6J*129S3 blastocysts. The chimeras were crossed with C57BL/6J mice, and germline transmitted mice were obtained. Mutant mice (Sec15b flox/+) carrying the floxed Sec15b allele were crossed with EIIa‐Cre mice (ubiquitous Cre activity). Because of the mosaic activities of Cre recombinase, the first offspring of the EIIa‐Cre; Sec15bflox/+mice might be chimeric with different deletions. Therefore, the chimeric offspring were backcrossed with C57BL/6J to generate Sec15b^+/−^ mice that were then intercrossed to produce Sec15b‐deficient mice (Sec15b^−/−^). The primers used for mouse genotyping are as follows: EXOC6B‐F (5’‐CTAGCCAGCTTGCCTGTGATT‐3') and EXOC6B‐R (5'‐ AGAGCCAGATGAACGGAACCT‐3'). A 422‐bp or a 905‐bp fragment was produced from the null allele or the wild‐type allele (Figure S6A,B, Supporting Information). All animal protocols were approved by Shanghai Biomodel Organism Science & Technology Development Co. Ltd.

### Generation of STAT3‐R31K Mutant Mouse

The BAC clone containing the STAT3 R31K gene (bMQ‐338j08, bMQ‐356g21, bMQ‐386p20, Source Bioscience) was identified in the mouse BAC (bMQ) Library. The plasmid PL‐452 and strain EL‐350 were provided by Liu Pengtao (Wellcome Trust Sanger Institute, Cambridge, UK). The plasmid pSC101‐BAD‐γβα‐A‐tet was provided by Zhang Youming (Gene Bridges GmbH, Germany). DH5α was obtained from Biotake, and pBR322‐MK was modified by the Shanghai Model Organisms Center.

The mouse STAT3‐001 transcript consists of 24 exons, with the mutation site located in exon 2. Neo and frt elements were placed in the intron, and a point mutation was introduced to generate the R31K mutation. The targeting vector for the CG to AA mutation in exon 2 of STAT3 was constructed using ET cloning. The procedure was as follows: Using the BAC plasmid as the template DNA, four short homology arms (A, B, C, D) were amplified by PCR, which was then knocked into the retrieve plasmid by homologous recombination, forming the vector pBR322‐MK‐STAT3 R31K‐KI. The construct was verified by restriction enzyme digestion and sequencing. Then the vector was electroporated into ES cells, followed by positive and negative drug selection. Double‐resistant cell clones were picked and cultured, and PCR was used to identify clones that underwent homologous recombination. ES cells with confirmed homologous recombination were injected into blastocysts and transferred into embryos. Finally, chimeric mice were bred, and their genotypes were confirmed by PCR. All animal protocols were approved by Shanghai Biomodel Organism Science & Technology Development Co. Ltd.

The primers used for construction of the targeting vector are as follows, Stat‐A‐F: ACGCGTCGACTCTTAAATCAGTTTCTA, Stat‐A‐R: CGGGGTACCGCTAAAAGCAAAGCGCAG; Stat‐B‐F: GGGGTACCATCGTGGCCCGATGCCTG, Stat‐B‐R: CGCGGATCCACCTCAACTCCCAGCCCT; Stat‐C‐F: CGATATCGAGACCCCTGACTGCAGC, Stat‐CM‐R: GGAACTGCttCAGCTCCATGGGG, Stat‐CM‐F: CATGGAGCTGaaGCAGTTCCTGGC; Stat‐C‐R: GGAATTCGAGACTAAGTTACCTCTG; Stat‐D‐F: GGAATTCGGGATCCAACGCATCTGTTCACAG, Stat‐D‐R: CGTCGACGTGGTAGACCCCAATTTCTACC. Primers for ES cell genotyping are as follows: ES‐5‐up: ACACTACCCAGCCATAGCACGTAG, ES‐5‐low: CTGAGCCCAGAAAGCGAAGGA; ES‐3‐up: CCTCCCCCGTGCCTTCCTTGAC, ES‐3‐low: TCAGCTATGCATAGCAAGAACCCG. Primers for mouse genotyping are as follows: P1‐F: TGGTGGTAGGATTTGTGAA, P2‐R: ACGCAGATAGGGCTGACTT; P3‐Neo‐R: GCTTGGCTGGACGTAAACTC.

### Generation of STAT3‐K177K180R Mutant Mouse

STAT3‐K177K180R (double KR mutation) homozygous mice were prepared using CRISPR/Cas9 genome editing system. First, guide‐RNA was designed following the STAT3 gene sequence information (http://www.IFformatics.jax.org /marker/MGI: 103038). Two active guide RNA were screened out once the guide‐RNA activity was detected: #1: gcttgcCTCCTTGGCTCTTG AGG and #2: TACAAAACCCTCAAGAGCCA AGG.

The effective guide‐RNA and Cas9 were transcribed into mRNA in vitro. In vitro transcription of Cas9 mRNA was performed using mMESSAGE mMACHIFE®T7 Ultra Kit, in vitro transcription of Guide RNA was performed by MEGAshortscript™ Kit, the products were purified by using MEGAshortscript™ Kit. Guide‐RNA.Cas9 mRNA and the recombinant donor DNA were injected into the male pronucleus of the zygotes by means of the micro injection, the donor fertilized eggs were obtained from the C57BL/6J mice. The sequence of donor DNA was as follows: GATCTAGAACAGAAAATGAAGGTGGTGGAGAACCTCCAGGACGACTTTGATTTCAACTACAgAACCCTCAgGAGCCAAGGAGgcaagcgagctgtggggacggggcagtctgtgcaagg.

The positive F0 generation mice were selected and intercrossed with wild type C57BL /6J mice. The primers used for detecting offspring genotype were as follows: *STAT3*‐K177/180R‐1 (5'‐CAGAGAAGCAGCAGATGTTGGAG‐3') and *STAT3*‐K177 /180R‐2 (5'‐ATGGTTGTAGGCTCACGGTCAG‐3'). A250‐bp PCR product was produced and then sequenced. The wild type mice and K177/180R homozygous mice genotyping were as follows, the site mutation of K177/180R heterozygous mice appeared in the form of overlapping peaks in the sequence diagram (Figure S5A, Supporting Information), Wt: TACAAAACCCTCAAGAGCC and Mut: TACAgAACCCTCAgGAGCC.

### Measurement of Various Indexes in STAT3‐K177K180R Mutant Mice

The routing blood test in mice (WT, heterozygous, and homozygous) were detected with automated hematology analyzer (HITACHI 7020 713‐0002). At 4–5 weeks of age, the mice (Wild Type, heterozygous, and homozygous) were weighed, and one homozygous mouse was used as the base 1. The weight of the other mice in the same nest was divided by the weight of this homozygous mouse, and the relative weight was obtained. WT, heterozygous, and homozygous mouse embryos of 16 days were used for skeletal imaging analysis with microscope CT with SKYSCAN 1176 device or for Alcian Blue‐Alizarin Red staining. All the blood samples were stained for RBC with Wright's staining solution (Nanjing Jiancheng Bioengineering Institute, D007).

### RNA‐Seq Measurement

Total RNA from embryos (12 days) of STAT3*^mut/mut^* and STAT3*^wt/wt^* mice which come from the same womb were extracted using TRIzol reagent (Invitrogen) following the manufacturer's protocols. RNA quantity, quality and integrity were analyzed with NanoDrop ND‐1000 and standard denaturing agarose gel electrophoresis. Sample labeling and array hybridization were performed according to the Agilent One‐Color Microarray‐Based Gene Expression Analysis protocol of Agilent Technology. The Whole Mouse Genome Oligo Microarray (Agilent 4*44k) represents all known genes and transcripts in the mouse genome (Kyoto Encyclopedia of Genes and Genomes, KEGG). All the microarray experiments were performed by Kangchen Biotechnology Limited (Shanghai, China). The accession number for all raw sequencing data is available at GEO: GSE271656.

### Flow Cytometry Test

Peripheral blood from 19‐week‐old STAT3 K177/180R mutant mice (homozygous, heterozygous, and wild‐type) was isolated and subjected to flow cytometry analysis to detect the levels of Th17 and Treg cells. The kits used for flow cytometry analysis were the Mouse Regulatory T Cell Staining Kit (KTR201, MultiSciences Biotech Co., Ltd) and the Mouse Th17 Staining Kit (KTH217, MultiSciences Biotech Co., Ltd).

### Statistics and Reproducibility

All experiments were repeated at least triple. N indicates the number of independent experiments performed. Data were expressed as Mean ± standard error of the mean (SEM). Statistical analyses were performed using Prism 8.0 software (GraphPad), pair wise statistical significance was evaluated by two‐tailed Mann–Whitney U‐test or Student's t‐test. Statistical significance between multiple groups was evaluated by two‐way ANOVA with Fisher's LSD test or Bonferroni test for multiple comparisons. *p* values ≤ 0.05 were considered statistically significant.

## Conflict of Interest

The authors declare no conflict of interest.

## Author Contributions

Y.E.C. and Y.F. conceived the project. L.X. and J.J. designed and validated the interaction of Sec15b and STAT3 and detected the phenotype of STAT3*^mut/mut^* and Sec15b^−/−^ mice. L.W. detected distribution of STAT3 during preimplantation mouse development. J.P., M.X., and C.Z. prepared samples for Sec15‐shRNA and RNA‐seq, and performed the in vitro experiments of CGR8 cells. Y.G. and G.X. conducted mouse breeding and cell culture. X.W, C.W, Y.E.C., and Y.F. discussed and interpreted the results. L.X. and Y.E.C. wrote the manuscript.

## Supporting information



Supporting Information

## Data Availability

The data that support the findings of this study are available in the supplementary material of this article.
