# Supplementary material for: LIF Promotes Sec15b‐Mediated STAT3 Exosome Secretion to Maintain Stem Cell Pluripotency in Mouse Embryonic Development
Source: Adv Sci (Weinh). 2024 Oct 30;11(48):2407971. doi: 10.1002/advs.202407971 (PMC11672271; doi:10.1002/advs.202407971)
Supplement: Supplementary file 1 — Supporting Information [file ADVS-11-2407971-s001.docx]

**LIF promotes Sec15b-mediated STAT3 exosome secretion to maintain stem cell pluripotency in mouse embryonic development**

Li Xu**^†^**^,1^, Jinjun Ji**^†^**^,1^, Lingbo Wang^3^, Jieli Pan^1^, Mingzhe Xiao^4^, Chenxi. Zhang^4^, Yihong Gan^1^, Guanqun Xie^1^, Mingdian Tan^4^, Xinchang Wang^5^, Chengping Wen^1^, Yongsheng Fan**^1,5^, Y. Eugene Chin*^2,4^

*^1^* *College of Basic Medical Science, Zhejiang Chinese Medical University, 548 Binwen Road, Hangzhou 310051, China*

*^2^**Institutes of Biology and Medical Sciences, Soochow University Medical College, Ren’ai Road 199, Suzhou, Jiangsu 215123, China*

*^3^Group of Epigenetic Reprogramming, State Key Laboratory of Cell Biology, Institute of Biochemistry and Cell Biology, Shanghai Institutes for Biological Sciences, Chinese Academy of Sciences, Shanghai 200031, China*

*^4^Key Laboratory of Stem Cell Biology, Institute of Health Sciences, Chinese Academy of Sciences, Shanghai 200031, China*

*^5^Department of Rheumatology, The Second Affiliated Hospital of Zhejiang Chinese Medical University, Hangzhou 310005, China*

**
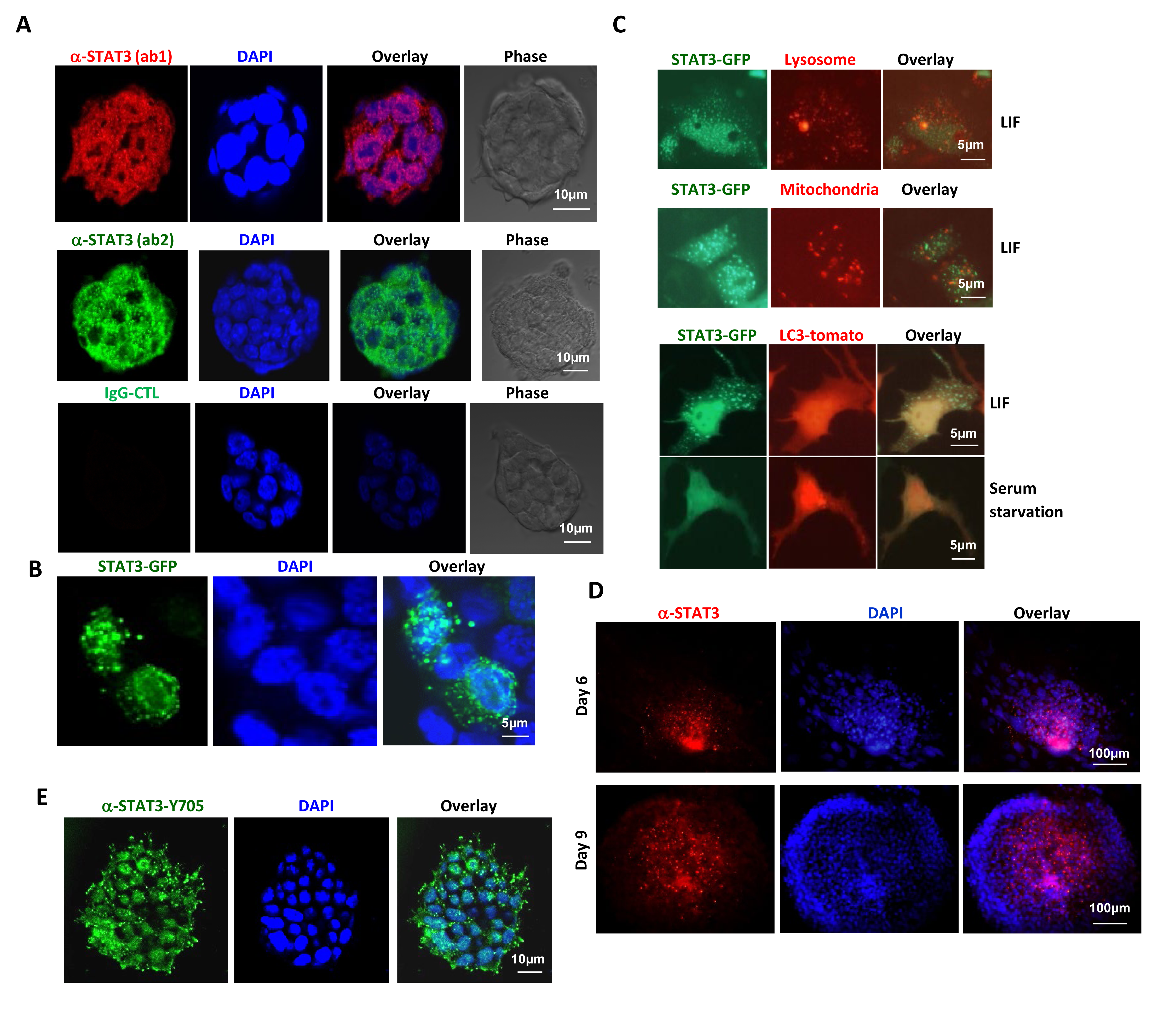
**

**Figure S1. STAT3 translocates into MVEs upon LIF stimulation.**

1. Confocal microscopy of endogenous STAT3 immuno-fluorescently stained with two different STAT3 antibodies (red, green) and DAPI (blue) in a single sphere of the CGR8 mESC maintained in LIF.
2. Confocal microscopy of expression of GFP-STAT3 in CGR8 cells maintained in LIF with DAPI nuclear staining.
3. GFP-STAT3 overexpression HEK-293T cells were treated with LIF for 30 min followed by staining with specific probes (red) to reflect subcellular organelles including lysosome, mitochondria, and LC3-tomato labelled autophagosomes.
4. Immunostaining of STAT3 and DAPI nuclear staining in day-6 and day-9 outgrowths during mESC derivation.
5. Confocal microscopy of endogenous STAT3 immunofluorescence stained with STAT3-pY705 antibody in a single sphere of the CGR8 maintained in LIF.


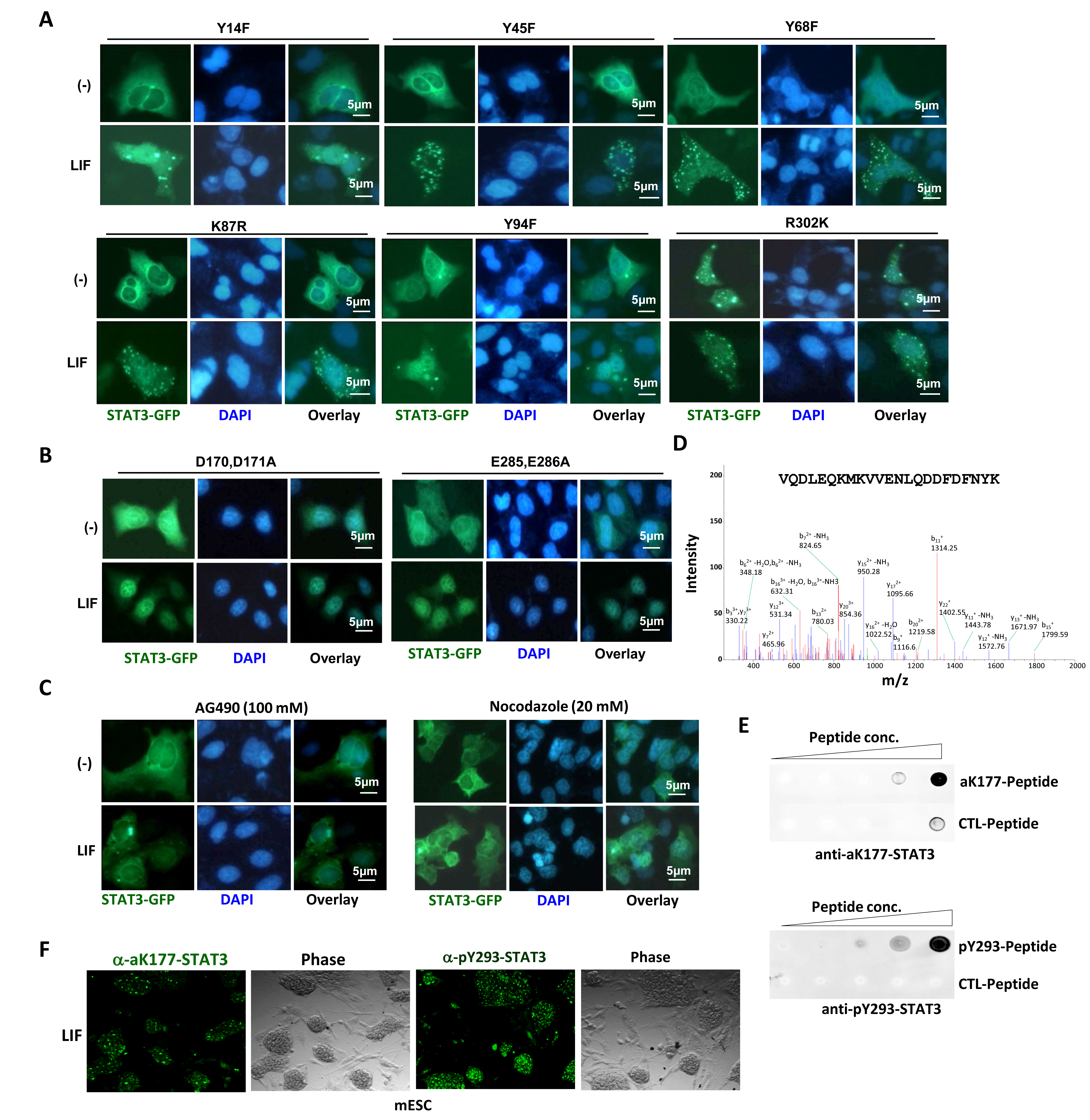


**Figure S2. Charged residues of the N-terminal domain of STAT3 are involved in MVEs translocation.**

1. The effect of different GFP-STAT3 site mutations of modifiable residues on STAT3 MVE formation.
2. The effect of GFP-STAT3-D170D171A and E285E286A mutations on STAT3 MVE formation.
3. GFP-STAT3 MVB formation in HEK293T cells growing in the medium supplemented with different inhibitors as indicated for 8 hrs followed by with or without LIF treatment for additional 30 min.
4. Mass spectrum of STAT3-aK177 peptide.
5. Purified polyclonal antibodies against STAT3-aK177 and STAT3-pY293 were confirmed by slot-blot analysis with indicated peptides spotted on the nitrocellulose membrane.
6. Confocal images of the D3 mESC maintained in LIF. STAT3 was immunostained with STAT3-pY293 and STAT3-aK177 antibodies respectively.


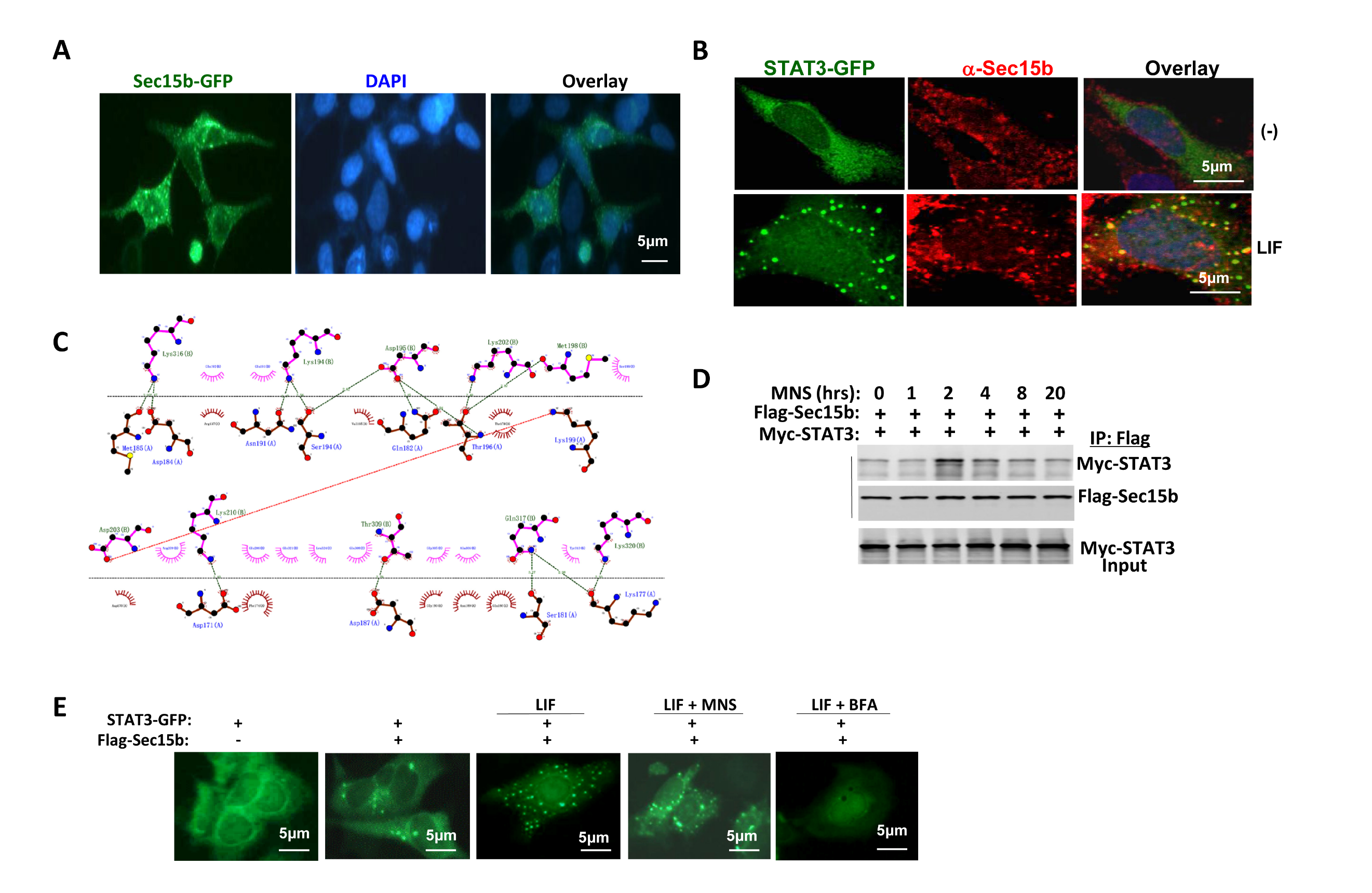


**Figure S3. STAT3 and Sec15b form complex and are colocalized in cells.**

1. HEK293T cells were transiently transfected with Sec15b-GFP and stained with DAPI for confocal visualization.
2. Co-localization of STAT3 and Sec15b in HEK293T cells was confirmed by GFP-STAT3 expression and anti-Sec15b immune-staining (red). HEK293T cell nuclei were stained with DAPI (blue).
3. Analysis of the two-dimensional interactions between STAT3 and Sec15b. 'A' represents STAT3 and 'B' represents Sec15b, with red dashed lines indicating salt bridges and green dashed lines representing hydrogen bonds.
4. HEK293T cells were transfected with Myc-STAT3 and Flag-Sec15b and treated with MNS for indicated times. Flag immune-precipitates were analyzed with anti-Flag for Sec15b and anti-Myc for STAT3.
5. HEK293T cells were transfected with STAT3-GFP and Flag-Sec15b and treated as indicated. STAT3 MVBs were visualized with confocal fluorescent microscope.


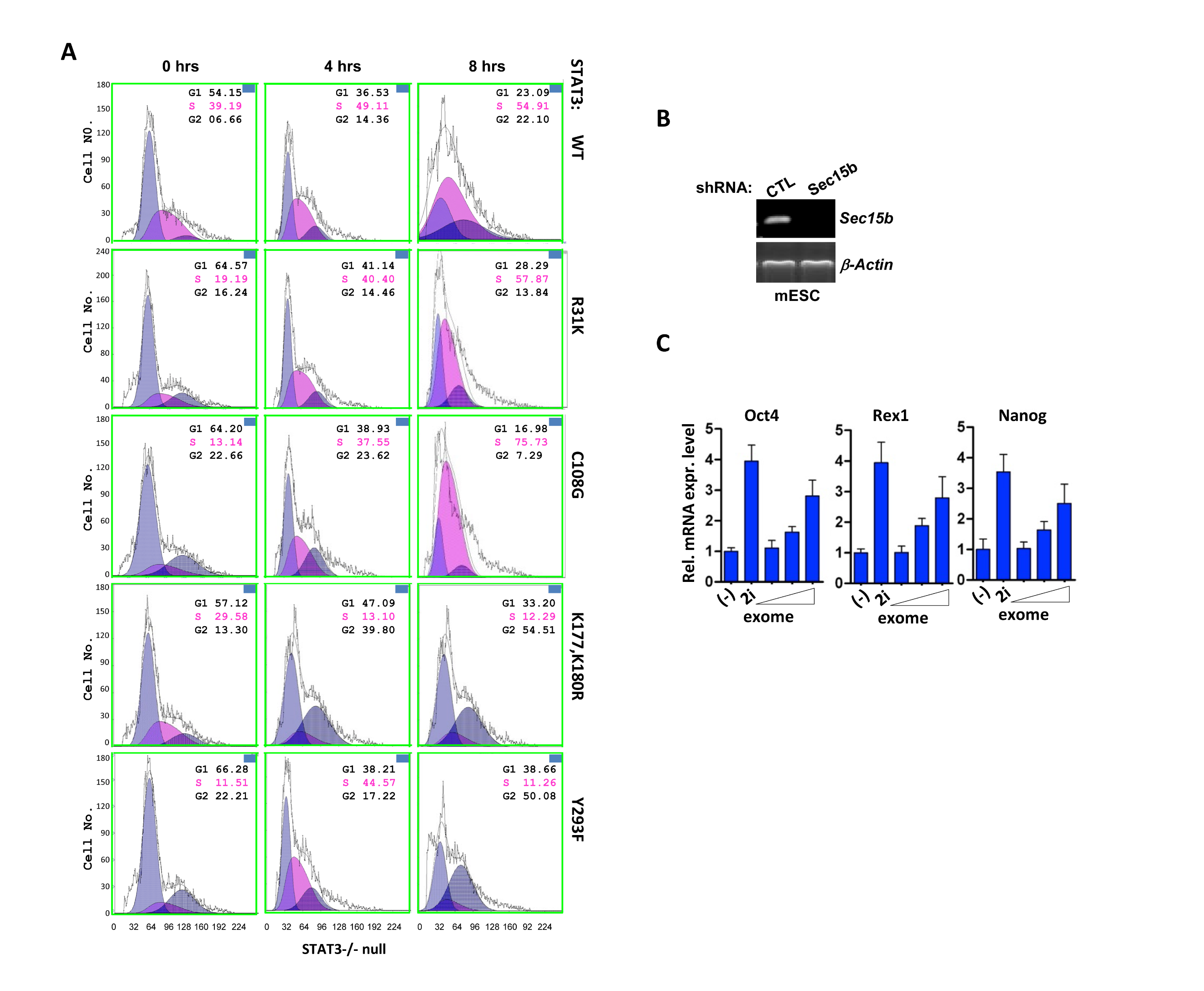


**Figure S4. STAT3 exosomes promote cell cycles.**

1. Representative assessment of cell cycles of the STAT3^-/-^ MEFs transfected with STAT3 variants as indicated. After serum starvation for 12 hrs, the cells were treated with LIF and serum for indicated times followed by FACS analysis. Cells in G1, S (pink color), and G2 phases were indicated.
2. Sec15b depletion in the mESC was confirmed by RT-PCR.
3. E14 mESC, maintained in LIF-free medium overnight, were treated with Smith 2i or increasing amount of STAT3-exosome for 24 hrs. Expression of the pluripotent genes was analyzed by qPCR.

**
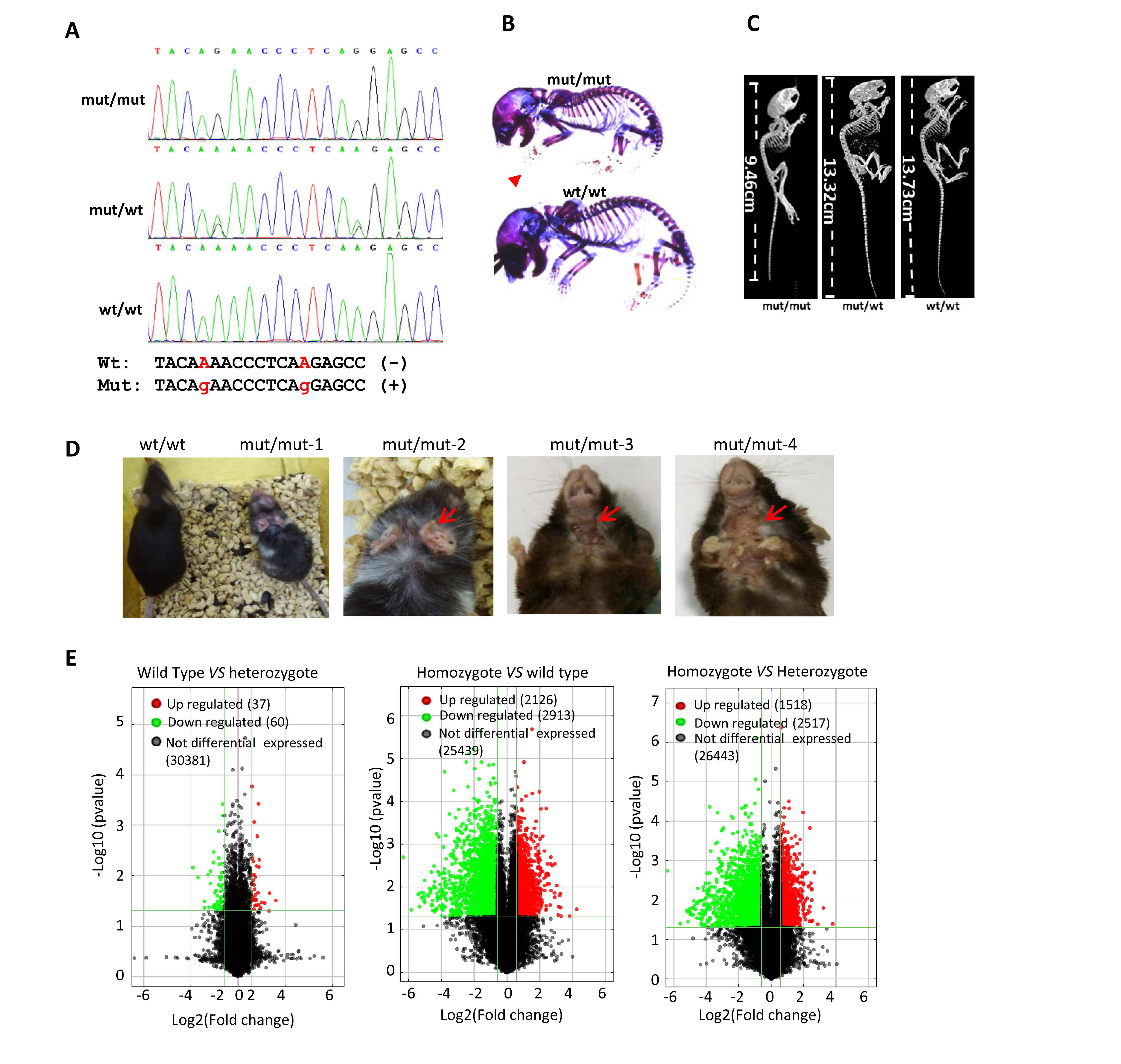
**

**Figure S5. Identification and analysis of STAT3*^mut/mut^* mice**

1. The genotypes of offspring from STAT3*^mut/wt^* intercross were identified by sanger sequencing.
2. The Alcian Blue - Alizarin Red Staining of 16 days of STAT3*^mut/mut^*, STAT3*^mut/wt^* and STAT3*^wt/wt^* embryo skeletons.
3. The skeletal imagings of 16 days of STAT3*^mut/mut^*, STAT3*^mut/wt^* and STAT3*^wt/wt^* embryos were observed by microscope CT with SKYSCAN 1176 device.
4. Changes on skin and hair of STAT3*^mut/mut^* mice
5. Volcano plot of differentially expressed genes among mouse embryos of STAT3*^mut/mut^* , STAT3*^mut/wt^*, and STAT3*^wt/wt^* mice


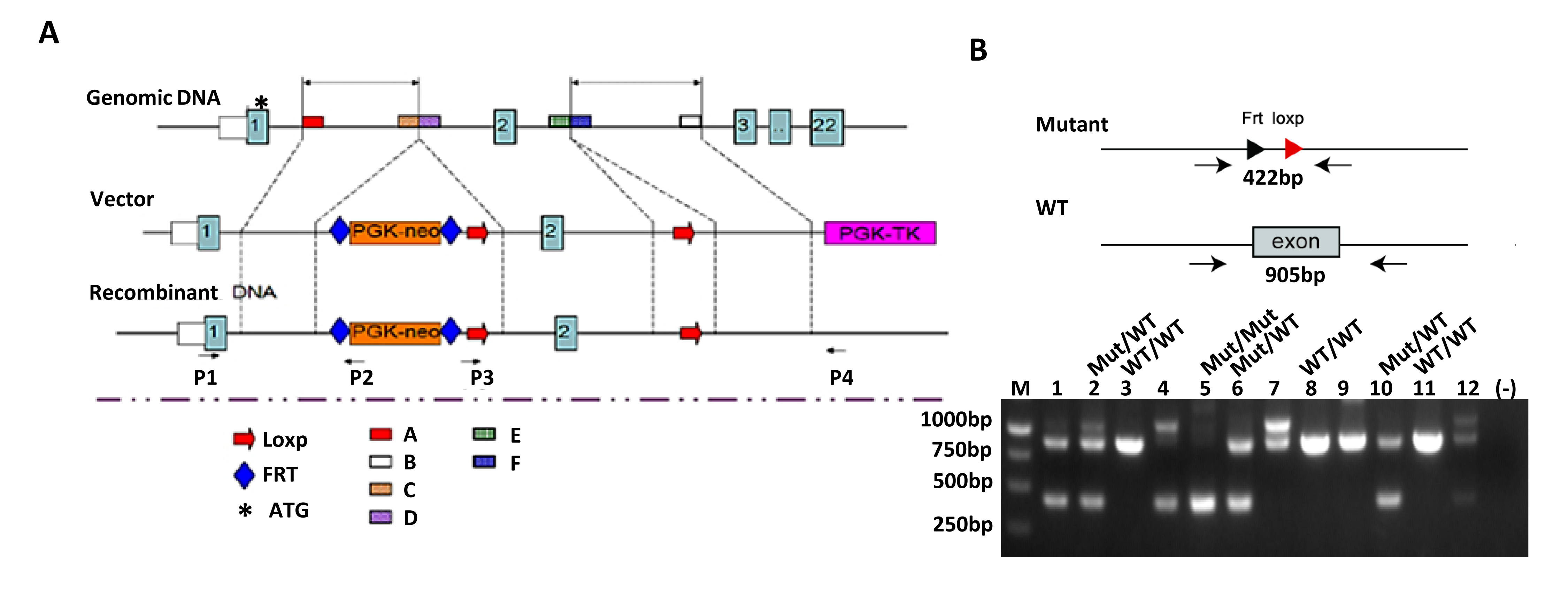


**Figure S6. Sec15b^-/-^ mice are constructed.**

1. The strategy for targeted inactivation of the Sec15b gene.
2. PCR genotyping of Sec15b^+/-^intercross offspring.


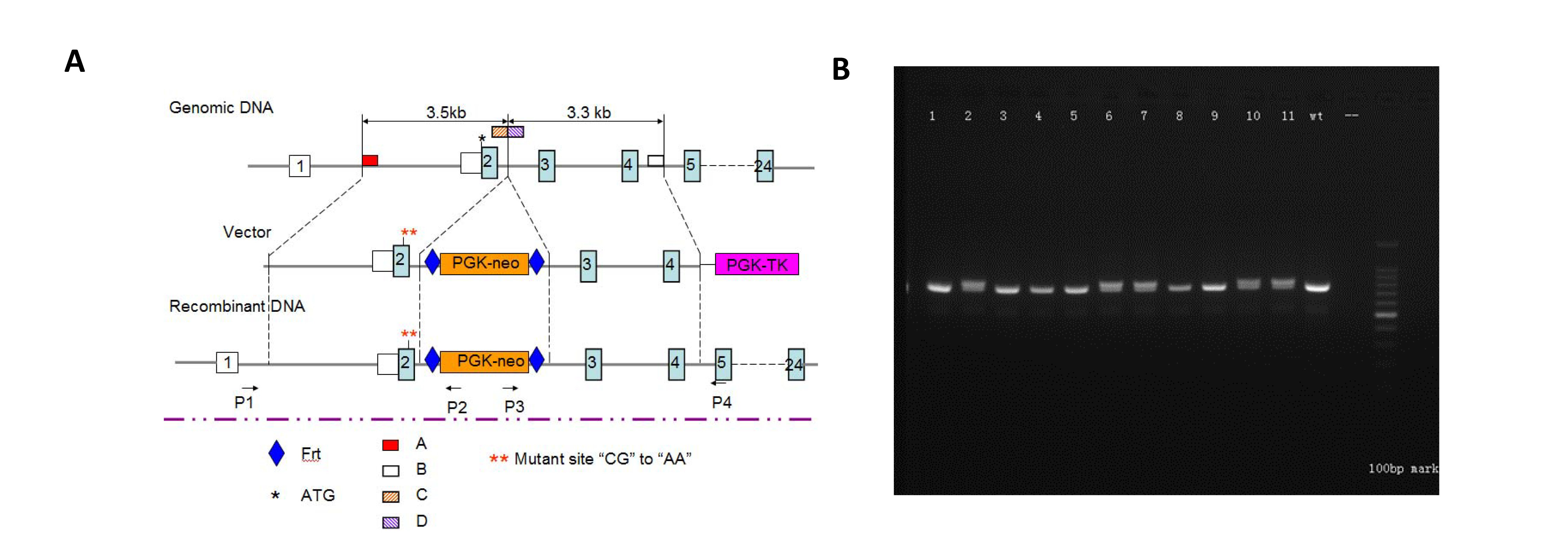


**Figure S7. STAT3-R31K mutant mice are constructed.**

(A) The strategy for targeted STAT3-R31K mutant.

(B) PCR genotyping of STAT3-R31K^+/-^ intercross offspring. PCR genotyping revealed that the offspring were either heterozygous or wild-type, with no homozygous mice identified.

**Table S1. Primers used for STAT3-domain, STAT5a-domain, and STAT3/STAT5a swab construction.**

| **ID** | **Primer Name** | | | **Sequence(5'to3')** |
| --- | --- | --- | --- | --- |
| 1 | | Stat3-1-F | TTGAATTCGATGGCTCAGTGGAACCAGCT | |
| 2 | | Stat3-335-R | CGgtcgacACCAGTCTTGATGACTAAGGG | |
| 3 | | Stat3-335-F | CGgtcgaccccttagtcatcaagactggt | |
| 4 | | Stat3-770-R | CGgggcccTCACATGGGGGAGGTAGCACA | |
| 5 | | Stat3-1-F | TTGAATTCGatggcgggctggattcaggcc | |
| 6 | | Stat3-338-R | CGgtcgacCTGGGTCTTCAGGACCTGAGG | |
| 7 | | Stat3-338-F | CGgtcgaccctcaggtcctgaagacccag | |
| 8 | | Stat3-797-R | CGgggcccCTACAACTGACGTGGGCTCCT | |
| 9 | | Stat-338-F stop | CGgtcgacTCACTGGGTCTTCAGGACCTGAGG | |
| 10 | | Stat3-131-F | CGgtcgaccaagggggccaggccaaccac | |
| 11 | | Stat3-131-R | CGgtcgac GTGGTTGGCCTGGCCCCCTTG | |
| 12 | | Stat3-335-R stop | CGgtcgac TCAACCAGTCTTGATGACTAAGGG | |
| 13 | | Stat5-127-F | CGgtcgac cgcgaagccaacaattgcagc | |
| 14 | | Stat5-127-R | CGgtcgac GCTGCAATTGTTGGCTTCGCG | |
| 15 | | Stat3-1-465-F | TTGAATTCGATGGCTCAGTGGAACCAGCT | |
| 16 | | Stat3-1-465-R | TAGGGCCCTCAGGAGATCACCACAACTGGC | |

**Table S2. Primers used for Sec15b cloning and truncated Sec15b construction.**

| **ID** | **Primer Name** | **Sequence(5'to3')** |
| --- | --- | --- |
| 1 | EcoRV-Sec15b-F | CGAGATATCTATGGAGCGGGGTAAGATG |
| 2 | NotI-Sec15b-R | CGAGCGGCCGCTCATGAGTGGTGGCTGCTGATGA |

**Table S3. Primers used for RT-PCR.**

| **ID** | **Primer Name** | **Sequence(5'to3')** |
| --- | --- | --- |
| 1 | Nanog-F | AGGGCTATCTGGTGAACG |
| 2 | Nanog-R | AGGAACCTGGCTTTGC |
| 3 | OCT4 -F | GAGGAAGCCGACAACAA |
| 4 | OCT4-R | GCTTCCTCCACCCACTT |
| 5 | Klf4-F | AAGGAAGCCCAGACGG |
| 6 | Klf4-R | GCAGGAAAGGAGGGTAGTT |
| 7 | Myc-F | ACAGCCACGACGATGCC |
| 8 | Myc -R | CGTTGAGCGGGTAGGGA |
| 9 | Stat3-F | TCTGGCTAGACAATATCATCG |
| 10 | Stat3-R | TACCTGGGTCGGCTTCG |
| 11 | Sec15b-F | AAATGAGCGAGATAAGCAGA |
| 12 | Sec15b-R | AACCAGCAAGGCAGATAGA |
| 13 | Actin-F | ACTGCCGCATCCTCTTCCTC |
| 14 | Actin-R | GGACTCATCGTACTCCTGCT |

**Table S4. Primers used for STAT3 site-directed mutagenesis**

| **ID** | **Primer Name** | **Sequence(5'to3')** |
| --- | --- | --- |
| 1 | Y14F-F | gacacacgcttcctgaagcagc |
| 2 | Y14F-R | GCTGCTTCAGGAAGCGTGTGTC |
| 3 | R31K-F | ccatggagctgaagcagttcctggc |
| 4 | R31K-R | GCCAGGAACTGCTTCAGCTCCATGG |
| 5 | Y45F-F | agactgggcatttgcagcca gc |
| 6 | Y45F-R | GCTGGCTGCAAATGCCCAGTCT |
| 7 | Y68F-F | tgaccagcaatttagccgattcc |
| 8 | Y68F-R | GGAATCGGCTAAATTGCTGGTCA |
| 9 | R70K-F | gcaatatagcaaattcctgcaa |
| 10 | R70K-R | TTGCAGGAATTTGCTATATTGC |
| 11 | R84/85K-F | cacaaccttaaaaaaatcaagc ag |
| 12 | R84/85K-R | CTGCTTGATTTTTTTAAGGTTGTG |
| 13 | K87A-F | gaagaatcgcgcagtttctgca |
| 14 | K87A-R | TGCAGAAACTGCGCGATTCTTC |
| 15 | K87R-F | gaagaatccggcagtttctgca |
| 16 | K87R-R | TGCAGAAACTGCCGGATTCTTC |
| 17 | R93K-F | tctgcagagcaagtatcttgagaa |
| 18 | R93K-R | TTCTCAAGATACTTGCTCTGCAGA |
| 19 | Y94F-F | cagagcaggtttcttgagaagc |
| 20 | Y94F-R | GCTTCTCAAGAAACCTGCTCTG |
| 21 | R107K-F | gatcgtggccaaatgcctgtggg |
| 22 | R107K-R | CCCACAGGCATTTGGCCACGATC |
| 23 | C108G-F | cgtggcccgaggcctgtggga ag |
| 24 | C108G-R | CTTCCCACAGGCCTCGGGCCACG |
| 25 | R114K-F | tgggaagagtctaagctcctccaga |
| 26 | R114K-R | TCTGGAGGAGCTTAGACTCTTCCCA |
| 27 | K140R-F | gtagtgacagagaggcagcagatgttg |
| 28 | K140R-R | CAACATCTGCTGCCTCTCTGTCACTAC |
| 29 | K140A-F | gtagtgacagaggcgcagcagatgttg |
| 30 | K140A-R | CAACATCTGCTGCGCCTCTGTCACTAC |
| 31 | K153R-F | aggatgtccggaggcgagtgcagga |
| 32 | K153R-R | TCCTGCACTCGCCTCCGGACATCCT |
| 33 | K153A-F | aggatgtccgggcgcgagtgcagga |
| 34 | K153A-R | TCCTGCACTCGCGCCCGGACATCCT |
| 35 | K161/163R-F | tagaacagagaatgagggtggtggaga |
| 36 | K161/163R-R | TCTCCACCACCCTCATTCTCTGTTCTA |
| 37 | K161/163A-F | tagaacaggcaatggcggtggtggaga |
| 38 | K161/163A-R | TCTCCACCACCGCCATTGCCTGTTCTA |
| 39 | D170A-F | cctccaggccgactttgatttcaa |
| 40 | D170A-R | TTGAAATCAAAGTCGGCCTGGAGG |
| 41 | D171A-F | cctccaggacgcctttgatttcaa |
| 42 | D171A-R | TTGAAATCAAAGGCGTCCTGGAGG |
| 43 | D170/171A-F | agaacctccaggccgcctttgattt |
| 44 | D170/171A-R | AAATCAAAGGCGGCCTGGAGGTTCT |
| 45 | D173A-F | caggacgactttgctttcaacta |
| 46 | D 173A-R | TAGTTGAAAGCAAAGTCGTCCTG |
| 47 | Y176F-F | tgatttcaacttcaaaaccctc |
| 48 | Y 176F-R | GAGGGTTTTGAAGTTGAAATCA |
| 49 | K177R-F | tcaactacagaaccctcaagagc |
| 50 | K177R-R | GCTCTTGAGGGTTCTGTAGTTGA |

**Table S5. Primers used for STAT3 site-directed mutagenesis**

| **ID** | **Primer Name** | **Sequence (5'to3')** |
| --- | --- | --- |
| 1 | K180R-F | acaaaaccctcaggagccaagga |
| 2 | K180R-R | TCCTTGGCTCCTGAGGGTTTTGT |
| 3 | K177/180R-F | tcaactacagaaccctcaggagccaa |
| 4 | K177/180R-R | TTGGCTCCTGAGGGTTCTGTAGTTGA |
| 5 | K177180A-F | tcaactacgcaaccctcgcgagccaa |
| 6 | K177/180A-R | TTGGCTCGCGAGGGTTGCGTAGTTGA |
| 7 | S181A-F | accctcaaggcccaaggagaca |
| 8 | S181A-R | TGTCTCCTTGGGCCTTGAGGGT |
| 9 | S181E-F | accctcaaggaacaaggagaca |
| 10 | S181E-R | TGTCTCCTTGTTCCTTGAGGGT |
| 11 | R214K-F | gaccagatgaagagaagcattgtg |
| 12 | R214K-R | CACAATGCTTCTCTTCATCTGGTC |
| 13 | Y230F-F | caatggagttcgtgcagaagac |
| 14 | Y230F-R | GTCTTCTGCACGAACTCCATTG |
| 15 | R245/246K-F | tgactggaagaagaagcagcagatcg |
| 16 | R245/246K-R | CGATCTGCTGCTTCTTCTTCCAGTCA |
| 17 | C251G-F | gcagatcgcgggcatcggaggccc |
| 18 | C251G-R | GGGCCTCCGATGCCCGCGATCTGC |
| 19 | C259G-F | tcccaacatcggcctggaccgtct |
| 20 | C259G-R | AGACGGTCCAGGCCGATGTTGGGA |
| 21 | R278K-F | aacttcagaccaagcaacaaatta |
| 22 | R278K-R | TAATTTGTTGCTTGGTCTGAAGTT |
| 23 | K282/283R-F | ccaacaaattaggagactggaggagc |
| 24 | K282/283R-R | GCTCCTCCAGTCTCCTAATTTGTTGG |
| 25 | E285/286G-F | taagaaactg ggggggctgc agcag |
| 26 | E285/286G-R | CTGCTGCAGCCCCCCCAGTTTCTTA |
| 27 | Y293F-F | agaaagtgtccttcaagggcgac |
| 28 | Y293F-R | GTCGCCCTTGAAGGACACTTTCT |
| 29 | D296A-F | tacaagggcgcccctatcgtgca |
| 30 | D296A-R | TGCACGATAGGGGCGCCCTTGTA |
| 31 | R302K-R | tcgtgcagcacaagcccatgctgg |
| 32 | R302K-F | CCAGCATGGGCTTGTGCTGCACGA |
| 33 | R382W-R | tcagagggtcttggaaatttaacat |
| 34 | R382W-F | ATGTTAAATTTCCAAGACCCTCTGA |
| 35 | R423Q-R | ggaatggaggccaggccaattgtga |
| 36 | R423Q-F | TCACAATTGGCCTGGCCTCCATTCC |
| 37 | 170-180del-R | gtggagaacctccaggacagccaaggagacatgcag |
| 38 | 170-180del-F | CTGCATGTCTCCTTGGCTGTCCTGGAGGTTCTCCAC |
| 39 | 160-180del-R | gtgcaggatctagaacagagccaaggagacatgcag |
| 40 | 160-180del-F | CTGCATGTCTCCTTGGCTCTGTTCTAGATCCTGCAC |
| 41 | 182-192del-R | tacaaaaccctcaagagccaggatctgaatggaaac |
| 42 | 182-192del-F | GTTTCCATTCAGATCCTGGCTCTTGAGGGTTTTGTA |
| 43 | 128-138del-R | gcagcccagcaagggggcccaacagccgccgtagtg |
| 44 | 128-138del-F | CACTACGGCGGCTGTTGGGCCCCCTTGCTGGGCTGC |
